# Development and validation of versatile species-specific primer assays for eDNA monitoring and authentication of 10 commercially important Peruvian marine species

**DOI:** 10.1371/journal.pone.0313181

**Published:** 2025-07-02

**Authors:** Alan Marín, Ruben Alfaro, Lorenzo E. Reyes-Flores, Claudia Ingar, Luis E. Santos-Rojas, Irina B. Alvarez-Jaque, Karen Rodríguez-Bernales, Cleila Carbajal, Angel Yon-Utrilla, Eliana Zelada-Mázmela

**Affiliations:** 1 Laboratorio de Genética, Fisiología y Reproducción, Facultad de Ciencias, Universidad Nacional del Santa, Chimbote, Perú; 2 Ecobiotech Lab S.A.C., Trujillo, Perú; UTRGV: The University of Texas Rio Grande Valley, UNITED STATES OF AMERICA

## Abstract

Molecular identification assays provide crucial support in the research and regulation of aquatic resources. Among them, species-specific primers provide significant discriminatory power for fast and simultaneous differentiation of closely related species. In this study, we developed species-specific primers for environmental DNA (eDNA) monitoring and identification of 10 fish and shellfish species commonly found in the Peruvian seafood sector. To ensure versatility and high specificity, our primers were subjected to various testing methods including PCR, qPCR, and DNA sequencing, supported by robust validation assays. This validation process included a) an *in-silico* stage using self-generated and public DNA sequences; b) an *in-vitro* stage using target species sourced from vouchered specimens, as well as fresh and cooked commercial samples, early life stages, and a wide range of non-target species; and c) an *in-situ* stage using eDNA samples collected from different Peruvian marine ecosystems. Our novel species-specific primers successfully passed the validation process, demonstrating high efficiency and specificity by unequivocally identifying all target species with 100% accuracy and without cross-species reactions. These primers are thus valuable tools for eDNA monitoring, seafood authentication, and combating illegal, unreported, and unregulated fishing. The assays presented in this study can support effective fishery management and conservation efforts not only in the Peruvian fishery sector but also in other countries where our target species are present or imported.

## 1. Introduction

Accurate taxonomic identification is crucial in various fields, including species management, biodiversity monitoring, food authentication, wildlife trafficking regulation, outbreak surveillance of pathogenic agents, and medical diagnosis [1–6], to mention a few. DNA sequencing is undoubtedly one of the most reliable and accurate methods for achieving species-level identification [7,8]. However, despite its high accuracy, DNA sequencing technologies remain time-consuming, labor-intensive, and relatively expensive [7,8].

A cheaper and faster molecular approach for accurately identifying species, even from highly degraded DNA, is based on species-specific primer (henceforth SSP) assays combined with Polymerase Chain Reaction (PCR) [9]. The application of SSPs for rapid species identification has gained significant attention from researchers, certification bodies, and law enforcement agencies due to their accuracy, time and cost-effectiveness, practical ease of use, and straightforward interpretation of results [9,10]. The effectiveness of SSPs relies on having a perfect or near-perfect complementarity between the primer and the target species’s template strand. Conversely, to minimize the risk of cross-species amplification, it is essential to introduce nucleotide mismatches within the hybridization region of non-target species, especially at the primer’s 3’ end (the last five nucleotides in that region) [11]. The most detrimental effects occur when there is a mismatch at the 3’-terminal position (the first nucleotide from the 3’ end), which can disrupt polymerase activity, greatly diminishing PCR efficiency or leading to a complete abolishment of PCR amplification [11,12].

Species-specific primers are designed to amplify specific short mitochondrial alleles, typically ranging from 100 to 300 bp, which are found in greater numbers per cell than nuclear genome copies [13,14]. Small-sized amplicons offer significant advantages for species identification in processed products through standard PCR [15–17]. It is also well suited for qPCR platforms, using different kinds of starting genetic material, including genomic DNA sourced from both fresh and processed organic tissues [18–20], early planktonic life stages [21], homogenized tissues or non-invasive DNA obtained by mucus swabbing, which can be amplified via direct qPCR without the need for a DNA isolation step [3,5,22]. Additionally, SSPs can be used with different sources of environmental DNA (eDNA), such as fresh and marine water environments [23–25], water from bags used for international fish transport [20], pond sediments [26], terrestrial soil [27], and airborne eDNA [28].

Species-specific eDNA detection is a fast, cost-effective, and highly sensitive technique that relies upon the presence of free DNA molecules shed by the organisms inhabiting a specific ecosystem [13]. The genetic material is usually collected through filtration and then PCR amplified using SSPs, allowing for the testing of the presence or absence of target species [29]. Species-specific eDNA validation typically involves three main steps: (1) *in-silico* validation stage using DNA sequences from target and non-target species for SSP design, (2) *in-vitro* validation stage by PCR amplification of tissue-derived DNA from target and non-target species, and (3) *in-situ* validation stage by PCR amplification of eDNA samples [13]. Currently, eDNA is successfully used worldwide to assess the presence or absence of invasive, endangered, or commercially important aquatic species, including fish, mollusks, crustaceans, amphibians, reptiles, and marine mammals [24,30]. Furthermore, several species-specific eDNA studies have reported strong correlations between target species abundance and eDNA concentrations in various aquatic organisms [31–35]. This demonstrates that eDNA not only accurately maps the distribution of target species but also serves as a promising tool for assessing fluctuations in stock biomass [36].

The Peruvian sea is home to a rich biodiversity [37–39], including at least 250 fish and 74 shellfish species that interact with the artisanal fishery [40]. Among the most sought-after and heavily exploited species are those from the families Centrolophidae (cojinovas), Epinephelidae (groupers), Haemulidae (grunts), Loliginidae (squids), Paralichthyidae (flounders), Pectinidae (scallops), and Serranidae (rock seabasses) [38]. Despite this abundance of seafood diversity, Peru lacks an official list of standardized commercial names for seafood species. Several studies have highlighted significant issues related to homonymy, where one name is used for multiple species (e.g., “*lenguado*” for various flatfish and “*mero*” for several grouper species) and synonymy, where multiple names refer to a single species (e.g., “*mocosa*”, “*cojinova mocosa*”, and “*ojo de uva*” all refer to the mocosa ruff, *Schedophilus haedrichi*) [38,41–44]. The extensive variety of seafood commercially available within the Peruvian fishery sector, combined with the use of vague or ambiguous commercial names and weak monitoring mechanisms throughout the supply chain, creates a conducive environment for unintended mislabeling or deliberate substitution of high-value species with cheaper alternatives [38,45].

The first peer-reviewed article—representing the most comprehensive study to date, featuring the broadest geographic coverage, the longest sampling period, and the highest number of DNA markers tested—to reveal seafood mislabeling along different Peruvian regions was recently published by Marín et al. [38]. This study covered the seafood supply chain from fish landing sites to wholesale markets, supermarkets, and restaurants in six regions: Tumbes, Piura, Lambayeque, La Libertad, Ancash, and Lima. The samples were collected from July 2016 to March 2018, and the results revealed that 26.72% of the samples collected in 5 out of the 6 surveyed regions were mislabeled. Subsequent peer-reviewed articles based on full DNA barcoding (COI) analyses reported even higher rates of mislabeling in the Peruvian seafood sector. For example, Biffi et al. [43] identified mislabeling rates of 32.7% in fish, squid, and a cetacean species from samples collected between May and June 2017. These samples were obtained from landing sites, wholesale and retail markets, supermarkets, and restaurants from Lima and Tumbes regions. Additionally, Velez-Zuazo et al. [46] reported a 43% mislabeling rate for fish samples (fresh, refrigerated, frozen, uncooked, and marinated) collected from September 2017 to February 2018 in wholesale fish markets, supermarkets, and restaurants in the Lima region.

Based on the evidence reported in the aforementioned studies, it is by all means clear that seafood mislabeling has emerged as a significant issue within different levels of the Peruvian supply chain. Seafood mislabeling affects conservation efforts, effective fishery management, and consumer finances. Hence, there is an urgent need to develop molecular assays for the rapid and accurate identification of seafood species to combat mislabeling. Despite that, only four marine species from Peru have been targeted for the development of SSPs including three fish [3,18] and one bivalve [15,16]. This study aimed to develop and evaluate the specificity, utility, and versatility of novel SSPs targeting 10 commercially important marine species. These novel SSP sets were validated through three different stages: 1) *in-silico*, using self-generated and publicly available DNA sequences; 2) *in-vitro*, using hatchery-reared larvae, vouchered specimens, and forensic samples collected from an aquaculture facility, landing sites, fish markets, supermarkets, and restaurants; and 3) *in-situ*, using eDNA samples collected from northern and central Peruvian marine ecosystems.

## 2. Materials and methods

### 2.1. Case study

A selected group of fish and shellfish species were selected as candidates for the development and evaluation of SSPs based on some of the following criteria: a) they are of high value or commercial interest, b) they are targets for substitution with cheaper species or are used to substitute other species, c) they are labeled with ambiguous commercial names, which may include species that share a name with two or more species, those labeled with a generic “umbrella” term, and species that are sold under several different names, and d) they have similar morphological characteristics, such as being congeneric or closely related species.

### 2.2. Biological samples collection and DNA extraction

#### 2.2.1. Fresh, processed, and cooked seafood samples collection and DNA isolation.

Fresh samples of target and non-target species were collected from September 2017 to July 2024 from local commercial divers, fish landing sites, wholesale fish markets, local markets, supermarkets, and restaurants from different coastal Peruvian regions including Tumbes, Piura, Lambayeque, La Libertad, Ancash, Lima, Pisco, and Tacna; and Santa Elena in Ecuador (Table 1 and S1 Table). No specific permits were necessary for the field sampling methods. The sampled locations are of public access and not protected in any way, and the collected specimens are not endangered or protected under any law. Processed seafood samples were collected from local supermarkets in Peru (frozen scallops and squids) and Japan (dried squids). For each target species, we collected a whole specimen that was used as a taxonomic voucher for further morphological identification by a specialist taxonomist and molecular analysis by DNA barcoding. Voucher specimens were deposited in the DNA barcoding sample collection of the Laboratory of Genetics, Physiology, and Reproduction of the National University of Santa (Ancash, Peru). Small and medium-sized species were collected as whole individuals, while fin or muscle tissues were sampled from large-sized specimens. Photograph records of whole specimens were taken for all collected samples. In some instances, we used tissue samples or archived DNA from our previous research or kindly donated by colleagues.

**Table 1:** Target shellfish and fish species used for the validation assays of the species-specific primers developed in this study.

Case number	Scientific name	Common name English/Spanish	Sampling site	Number of analyzed samples	Vouchered specimens (GenBank)/[BOLD] accessions	Collection date
1	*Argopecten purpuratus*	Peruvian scallop/ Concha de abanico	Sechura Bay (Piura, Peru)	10	(PP087160 to PP087173)	September 16, 2017
			Independecia Bay (Pisco, Peru)	10	(PP087174 to PP087193)	September 27, 2017
2	*Doryteuthis gahi*	Patagonian longfin squid/ Calamar común	Isla Tortugas (Ancash, Peru)	6	[PeMar_I0189 to PeMar_I0194, PeMar_I1983], (PQ459851, PQ459852)	June 6, 2017
			La Sirena market (Ancash, Peru)	1		September 17, 2018
			Modelo market (Tumbes, Peru)	14		February 1, 2019
3	*Paralichthys adspersus*	Fine flounder/ Lenguado fino	Buenos Aires market (Ancash, Peru)	2	(PP092941)	February 14, 2019
			Culebras, Huarmey (Ancash, Peru)	6		—
			Vila Vila (Tacna, Peru)	1		—
			Pacific Deep Frozen (Ancash, Peru)	11		July to September, 2021
4	*Etropus ectenes*	Sole flounder/ Lenguado boca chica	Caleta Grau fish landing site (Tumbes, Peru)	3	(PP092942)	February 2, 2019
			Caleta Grau fish landing site (Tumbes, Peru)	17		September 10, 2019
5	*Paralabrax callaensis*	Southern seabass/ Cabrilla fina	Modelo market (Tumbes, Peru)	4	(PP092943 to PP092947, PQ459826 to PQ459835)	February 1, 2019
			Cancas fish landing site (Tumbes, Peru)	5		February 1, 2019
			Buenos Aires market (Ancash, Peru)	11		March 24, 2019
6	*Paralabrax humeralis*	Peruvian rock seabass/ Cabrilla común	Modelo market (Tumbes, Peru)	7	(PP092948 to PP092952, PQ459836 to PQ459845)	February 1, 2019
			Máncora market (Piura, Peru)	10		February 4, 2019
			Mayorista market (La Libertad, Peru)	5		February 16, 2019
			Buenos Aires market (Ancash, Peru)	10		March 24, 2019
7	*Hemilutjanus macrophthalmos*	Grape-eye seabass/ Ojo de uva	José Olaya wholesale fish market (Piura, Peru)	2	(PP092953, PQ459810 to PQ459812)	April 28, 2019
			José Olaya wholesale fish market (Piura, Peru)	2		April 29, 2019
			José Olaya wholesale fish market (Piura, Peru)	2		May 8, 2019
			José Olaya wholesale fish market (Piura, Peru)	2		May 10, 2019
			Cabo Blanco fish landing site (Piura, Peru)	3		June 5, 2019
8	*Schedophilus haedrichi*	Mocosa ruff/ Cojinova mocosa	Mayorista market (La Libertad, Peru)	6	(PP092954, PQ459805 to PQ459809)	February 16, 2019
			Puerto Pizarro fish landing site (Tumbes, Peru)	1		March 16, 2019
			José Olaya wholesale fish market (Piura, Peru)	5		April 10, 2019
			Mayorista market (La Libertad, Peru)	2		April 11, 2019
			José Olaya wholesale fish market (Piura, Peru)	2		May 7, 2019
			José Olaya wholesale fish market (Piura, Peru)	5		May 8, 2019
			Santa Rosa wholesale fish market (Lambayeque, Peru)	3		May 9, 2019
9	*Alphestes immaculatus*	Pacific mutton hamlet/ Mero rojo	Santa Rosa wholesale fish market (Lambayeque, Peru)	12	(PP092955, PQ459846 to PQ459848)	March 25, 2019
10	*Anisotremus interruptus*	Burrito grunt / Chita dorada	José Olaya wholesale fish market (Piura, Peru)	8	(PP092956)	April 10, 2019
			José Olaya wholesale fish market (Piura, Peru)	6		May 7, 2019
			José Olaya wholesale fish market (Piura, Peru)	2		May 8, 2019

Genomic DNA from scallop samples was isolated from the adductor muscle using the automated DNA extractor iPrep TM (Invitrogen, Carlsbad, CA, USA). Genomic DNA from non-target bivalve, squid, and fish samples was isolated using the GeneJET Genomic DNA Purification Kit (Thermo Fisher Scientific, Carlsbad, CA, USA) or the standard phenol-chloroform protocol [47] from adductor muscle, tentacle tissues, or fin clips, respectively. DNA quantification was calculated using an Epoch spectrophotometer (BioTek Instruments, Winooski, VT, USA). Total gDNA was diluted to a final working concentration of 10–20 ng/μL and stored at −20 ˚C for further PCR analyses.

Commercially cooked samples of various presentations were collected from restaurants along the north-central Peruvian coast (Tumbes, Piura, Lambayeque, La Libertad, and Ancash regions) from May 2019 to April 2024. We targeted only seafood dishes whose labels covered the species of this study: scallops, squids, flounders, rock seabasses, eye-grape seabass, mocosa ruff, groupers, and grunts. Tissues were rinsed with distilled water and preserved in 96% ethanol at −20 ˚C for further DNA analysis. Genomic DNA from cooked samples was isolated using the standard phenol-chloroform protocol [47].

#### 2.2.2. Collection of early life stages of the fine flounder *Paralichthys adspersus* and DNA extraction.

To evaluate the performance of the SSPs in identifying fish species during early life stages, from July to September 2021 we collected premetamorphic larvae (13 days after hatching, hereafter DAH), metamorphic larvae (31 DAH), and postmetamorphic juveniles (54 DAH) of *Paralichthys adspersus* from an aquaculture farm facility (Pacific Deep Frozen) located in Huarmey (Ancash, Peru). Larva and juvenile specimens were cold-anesthetized and preserved in RNA*later* solution (Invitrogen, Carlsbad, CA, USA). Genomic DNA was isolated from a small piece of fin clip using the standard phenol-chloroform protocol [47].

#### 2.2.3. eDNA collection, filtration of water samples, and eDNA extraction.

**Fig 1:**
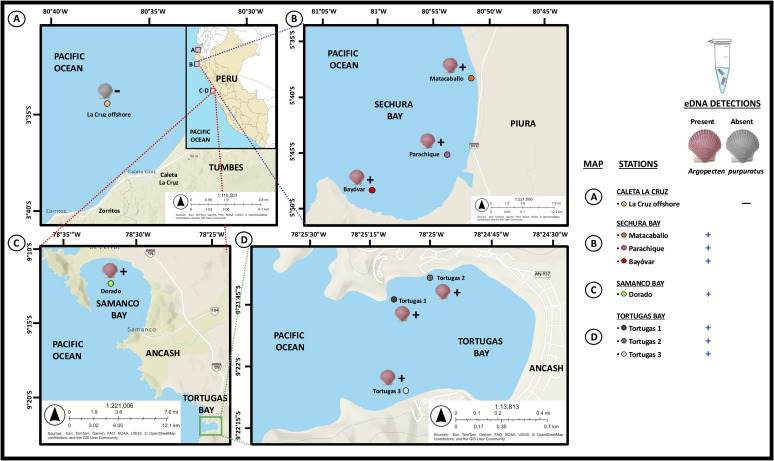
Map of the Peruvian eDNA sampling stations and eDNA detection results of *Argopecten purpuratus.* Map A: Caleta La Cruz sampling station (Tumbes region). Map B: Sechura Bay (Piura region) showing the Matacaballo, Parachique, and Bayóvar sampling stations. Map C: Samanco Bay (Ancash region) showing the El Dorado sampling station. Map D: Tortugas Bay (Ancash region) showing three sampling stations namely Tortugas 1, Tortugas 2, and Tortugas 3. Positive detections of *A. purpuratus* eDNA are depicted by a “+” sign and a colored scallop valve, while negative detections of *A. purpuratus* eDNA are depicted by a “-” sign and a scallop valve in black and white. The maps A, B, C, and D were reprinted from ArcGIS Online maps under a CC BY license, with permission from ESRI, original copyright 2025 Esri (The Outdoor Map World Edition basemap is supported by Esri, TomTom, Garmin, FAO, NOAA, USGS, © OpenStreetMap contributors, and the GIS User Community). The copyrights belong to ESRI, but according to the terms of use, the copyright holder does not need to apply for permission to use because it is free for academic publications, and can be used freely and commercially under the CC BY 4.0 license. The inset map in Map A was reprinted from MapChart Online maps under a CC BY license, with permission from MapChart, original copyright 2025. The copyrights belong to MapChart, but according to the terms of use, the copyright holder does not need to apply for permission to use because it is free for academic publications, and can be used freely and commercially under the CC BY 4.0 license.

*In-situ* eDNA validation assays should include eDNA samples from environments where the target species is present and also from environments where the target species is absent [13]. Therefore, we collected water samples in five field surveys (from October 2017 to may 2022) targeting seven different inshore stations belonging to two coastal bays with cultured and wild stocks of the Peruvian scallop *Argopecten purpuratus* (Sechura Bay in Piura region and Samanco Bay in Ancash region), a bay with only wild *A. purpuratus* (Tortugas Bay in Ancash region), and an offshore station out of the distribution range of *A. purpuratus* populations (La Cruz, Tumbes region) (Fig 1). The first field survey was performed in October 2017 at the Parachique station in Sechura Bay using 0.5 L new plastic containers and nitrocellulose filter membranes (0.45 μm pore size and 47 mm diameter), eDNA samples were collected from the bottom (5–6 m depth), midwater (2–3 m depth), and surface (0.5 m depth). The second field survey was carried out in February 2019 at Samanco Bay (Ancash) using 1 L sterilized laboratory glass bottles and nitrocellulose filter membranes (0.45 μm pore size and 47 mm diameter). eDNA samples were collected from the surface (0.5 m depth) water column. Samples from the third and fourth field surveys consisted of repurposed marine eDNA filters originally collected for a metabarcoding study targeting sponge-associated bacterial communities. Samplings were carried out from April to May 2022 at the Bayóvar and the Matacaballo stations in Sechura Bay (Piura region) and three stations from Tortugas Bay (Ancash region) using 1 L sterilized laboratory glass bottles and mixed cellulose ester (MCE) filter membranes (0.45 μm pore size and 47 mm diameter), eDNA samples were collected from the bottom water column (3–6 m depth, depending on each station) in close proximity to sponge communities. The field survey in the offshore location, namely La Cruz (Tumbes region), was performed in December 2017. eDNA samples were collected from the surface (0.5 m depth) water column using 1 L sterilized laboratory glass bottles and nitrocellulose filter membranes (0.45 μm pore size and 47 mm diameter). Seawater samples were filtered within 45 min of collection, except for the sample collected at the La Cruz offshore station, which was kept in a cooler box containing frozen gel packs and filtered upon 4 h of collection. All eDNA samples were filtered using a manual vacuum pump connected to a 500 mL magnetic filter funnel (Rocker, Rocker Scientific Company Limited, Taiwan). To avoid contamination during field samplings, all materials (cooler box, gel packs, water containers, tweezers) were soaked in 25% bleach for 30 min, rinsed with 70% ethanol and distilled water, and sterilized under UV light. Disposable nitrile gloves were used during all filtering steps and replaced by new ones before filtering a new eDNA sample. After each round of filtration and before filtering the next sampling station, all equipment, including the filtering funnel, was soaked in 25% bleach for at least 10 min and rinsed with 70% ethanol and distilled water. For each sampling site, blank control samples were obtained by filtering 1 L of distilled water. After the filtration was completed, filters were carefully folded using sterilized tweezers and stored in 2 mL microtubes containing 96% ethanol. All filters were transported to the laboratory in a cooler box containing frozen gel packs. Once at the lab, filters were immediately kept at −20 °C until DNA extraction.

All eDNA extractions (eDNA-containing filters and blank control samples) were performed in a clean laboratory completely separated from pre-PCR and PCR rooms, following strict decontamination procedures (disinfection with 10% bleach, 70% ethanol, and UV-light sterilization) and use of disposable materials. In order to prevent contamination, only aerosol barrier tips were used during eDNA extractions. Filter membranes were removed from the ethanol, cut in half, air-dried, and shredded into small pieces using a sterile scalpel blade. Only eDNA extractions from the Tortugas 1 and Tortugas 3 stations were performed using half and quarter filters. Briefly, each shredded half (or quarter) filter was placed in a 2 mL Eppendorf tube and 600 µL lysis buffer (10 mM Tris-HCl, 5 mM EDTA, 20 mM NaCl, 1% SDS) and 50 µL proteinase K (10 mg/ml) were added to each filter tube and incubated at 55°C for 3 h in a shaking incubator, tubes were vortexed every 15 min. Then, 600 µL phenol was added and mixed by inversion and incubated for 15 min at room temperature in a shaker (600 rpm). After that, 600 µL chloroform was added and mixed by inversion and incubated for 20 min at room temperature in a shaker (600 rpm). Samples were centrifuged at 13500 rpm at room temperature for 15 min. To precipitate eDNA, 500 µL of the aqueous phase was transferred to a clean 2 mL tube, mixed with 50 µL of 3M sodium acetate (NaOAc) and 1250 µL of 99% cold ethanol and incubated at – 20 °C overnight. Next, the microtube was centrifuged at 13500 rpm for 30 min at 4 °C, and the ethanol was poured out. The pellet was washed with 500 µL of 70% ethanol, centrifuging at 13500 × rpm for 5 min at 4 °C, the ethanol was removed and the pellet was dried at 37 °C for 7 min. Once dried, the pellet was dissolved in 50 µL 1 × TE Buffer pH 8.0 (Thermo Fisher Scientific Baltics, Vilnius, Lithuania).

### 2.3. Species authentication of voucher specimens and non-target species by DNA sequencing analysis

The species identity of 34 *A. purpuratus* and 27 *A. ventricosus* specimens was verified based on the DNA sequence of a partial fragment of the mitochondrial 16S rRNA gene (about 670 bp) amplified using a common *Argopecten* primer PurvenF developed herein and the universal primer 16Sbr-H [48] (S2 Table) under the following thermal cycling conditions: initial denaturation at 94 °C for 5 min, followed by 30 cycles of 94 °C for 20 s, 60 °C for 20 s, and 72 °C for 25 s, and a final extension step at 72 °C for 7 min. PCR reaction mixtures consisted of a 20 µL final volume containing 0.1 µL of Maximo *Taq* DNA Polymerase (GeneOn, GmbH, Nurnberg, Germany), 2.5 µL buffer 10 × , 1 µL dNTPs (2.5 mM), 0.08 µL each primer (50 µM), 0.38 µL MgCl_2_ (100 µM), 1 µL of template DNA (10 ng/µL), and 14.86 µL ultrapure H_2_O. Successful PCR amplification products were visualized in a 1.5% agarose gel (EMD Millipore, Billerica, MA, USA) electrophoresis, and amplicons were stained with GelRed Nucleic Acid Gel Stain (BIOTREND Chemikalien GmbH, Köln, Germany). PCR products were Sanger sequenced in both directions at Macrogen (Seoul, South Korea). All obtained scallop sequences were deposited in GenBank/DDBJ/EMBL DNA databases with accession numbers from PP087160 to PP087193 (*A. purpuratus*) and PP087194 to PP087220 (*A. ventricosus*).

Fish and squid voucher specimens of target and non-target species were morphologically identified by an expert taxonomist and by DNA barcoding assay using the universal primer sets 16Sar-L/16Sbr-H [48], FishF1/FishR1 [49], or LCO1490/HCO2198 [50] (S2 Table) with amplification conditions described by Marín et al. [38]. PCR products were purified using FastAP Thermosensitive Alkaline Phosphatase and Exonuclease I (Thermo Fisher Scientific, USA) following the manufacturer’s instructions and sequenced using the same primer set on an ABI PRISM 3500 Genetic Analyzer (Applied Biosystems, Hitachi, Foster City, CA, USA) using the BigDye terminator v3.1 Cycle Sequencing Kit (Applied Biosystems, Waltham, Massachusetts, USA) at the Laboratory of Genetics, Physiology, and Reproduction (National University of Santa, Peru). All obtained sequences were deposited in GenBank/DDBJ/EMBL DNA databases with accession numbers from PP092941 to PP092966, PQ459826 to PQ459855, and PV562158.

Identification of all DNA sequences at the species level was accomplished by using both the Barcode of Life Data System (BOLD, http://www.boldsystems.org) selecting “species level barcode records” database and Basic Local Alignment Search Tool (BLAST) on the National Center for Biotechnology Information (NCBI, http://www.blast.ncbi.nlm.nih.gov/Blast.cgi). The current accepted scientific names were checked in The World Register of Marine Species (WoRMS, available at http://www.marinespecies.org).

### 2.4. *In-silico* analysis for species-specific primer design

Target mitochondrial genes for the designing of SSPs were selected based on high interspecific variability reported in fish and shellfish species identification studies [15,38,51,52]. Three mitochondrial genes were selected: the *cytochrome oxidase subunit I* (COI), the *16S ribosomal RNA* (16S rRNA), and the *12S ribosomal RNA* (12S rRNA). Aiming to include as many DNA sequences as possible from target and non-target species, we used self-generated and reference sequences retrieved from BOLD and GenBank databases. All sequences were multialigned in MEGA 7 software [53] generating different multi-sequence matrices that were used for intra- and interspecific variation analyses of the putative SSPs. At least three SSP sets for each target species were designed using AlleleID 7.0 software (PREMIER Biosoft, USA) or manually by searching non-conserved homologous regions at the 3’ end (defined as the last 5 nucleotides from the primer’s 3’ end [11]) of the primer hybridization site of closely related (e.g., congeneric) and non-related non-target species to ensure the specific amplification only in target species.

All SSP sets were designed to yield amplicon lengths between 100 and 300 bp and to work at high annealing temperatures (≥ 60 °C) to ensure reaction astringency. Candidate oligos were tested *in-silico* for secondary structure formation (hairpins, homo and hetero-dimers) using the IDT OligoAnalyzer tool (available at <http://www.idtdna.com>) and The Sequence Manipulation Suite [54] (available at <http://www.bioinformatics.org/sms2/index.html>). Lastly, the specificity of the candidate oligos was analyzed in the NCBI GenBank database using Primer–BLAST [55] selecting “nr” non-redundant nucleotide database and organisms limited to Class Bivalvia (taxid:6544) in scallop primer analyses, the orders Myopsida (taxid:551347) and Oegopsida (taxid:34542) in squid primer analyses, and the Parvphylums Osteichthyes (taxid:7898) and Chondrichthyes (taxid:7777) in fish primer analyses. Any match to freshwater species and species not found in the Eastern Pacific was disregarded. All designed oligonucleotides were synthesized by Integrated DNA Technologies (IDT, Coralville, Iowa, USA) and Invitrogen (Carlsbad, CA, USA).

### 2.5. *In-vitro* validation assays of species-specific primers by standard PCR and qPCR

#### 2.5.1. Optimization and validation of standard PCR assays.

To evaluate the species specificity of each SSP set, we first determined the maximum optimal annealing temperature by performing a gradient annealing temperature analysis ranging from 60 to 64 °C. To determine the minimum primer concentration that resulted in a reliable and specific PCR product, concentrations between 100 and 500 nM were evaluated at 50 nM increments. The optimal annealing temperature and primer concentration were initially tested in 5 individuals of the target species. All PCR reactions were performed in a Veriti 96 Well thermal cycler (Applied Biosystems, Foster City, CA, USA) using the Maximo *Taq* DNA Polymerase 2x-preMix (GeneOn GmbH, Nurnberg, Germany), which includes ammonium sulfate ((NH_4_)_2_SO_4_) in its buffer system. The NH_4_ ions destabilize weak hydrogen bonds and mismatched bases present in non-target loci and primer dimers, enhancing the yield of specific PCR products [56]. The specificity of the newly designed primers was tested *in-vitro* using DNA extracted from all collected individuals of each target species (ranging from 11 to 32 specimens), where we expected to obtain a single PCR product of the expected size.

SSP validation assays for target shellfish species were performed against different non-target bivalve (7 species from 6 families) and squid (4 species from 2 families) species (S1 Table). The yield of artifacts resulting from the interaction between the SSP and endogenous control primer sets during the duplex PCR trials prevented us from including an internal control reaction during the specificity validation assays targeting the Peruvian scallop and the common squid against non-target species. We discarded the possibility of the presence of inhibitors or poor quality of non-target species’ DNAs because during species authentication analyses using the DNA barcoding approach we obtained abundant PCR products. Therefore, the specificity validation assays of *A. purpuratus* and *Doryteuthis gahi* were based on the total absence of PCR products in non-target species.

For SSPs validation against non-target fish species, a duplex PCR was standardized using the SSP set (COI or 16S rRNA gene, depending on species) in a single reaction with an internal endogenous control using the fish universal primers MiFish-U [51] (S2 Table) designed to amplify a small fragment (about 220 bp) of the mitochondrial 12S rRNA gene. In the absence of target DNA, non-target fish species (27 fish species from 19 families, S1 Table) were expected to produce only the universal 12S rRNA gene amplicon, resulting in the visualization of a single band in the agarose gel, while target species yielded two PCR products corresponding to the SSPs and the endogenous control bands. When possible, in order to validate the high specificity of our SSPs and to evaluate if they can be used to distinguish between closely related species, up to 22 individuals of non-target congeneric species were included in the PCR validation trials. PCR reactions were performed in a Veriti 96 Well thermal cycler (Applied Biosystems, Foster City, CA, USA) using Maximo *Taq* DNA Polymerase 2 × -preMix (GeneOn GmbH, Nurnberg, Germany). All PCR reactions were visualized using a 1.5% agarose gel (EMD Millipore, Billerica, MA, USA) electrophoresis, and amplicons were stained with GelRed Nucleic Acid Gel Stain (BIOTREND Chemikalien GmbH, Köln, Germany).

#### 2.5.2. Optimization and validation of qPCR assays.

The specificity and efficiency of the SSP set for the identification of the target scallop species (*A. purpuratus*) were additionally evaluated in qPCR assays performed on a LightCycler 480 II (Roche Diagnostics GmbH, Penzberg, Germany) and run in triplicate on 96-well reaction plates (Roche Diagnostics GmbH, Mannheim, Germany) following MIQE guidelines [57]. qPCR mix reactions and cycling protocols for the identification of *A. purpuratus* are shown in panel A of S1 Fig. The 2x SYBR Green I Master (Roche Diagnostics, Mannheim, Germany) kit was used in all experiments. A melting curve analysis was conducted from 60 °C for 1 min with a rate of 2.2 °C per s up to 95 °C with continuous acquisition. All reactions were performed using a designated set of micropipettes for qPCR use only. A positive control (20 ng of target species gDNA) and a no-template control (NTC: 2 µL ultrapure H_2_O instead of template DNA) reaction were included in each qPCR run. Primer specificity confirmation was checked by melting curve analysis showing single sharp peaks only in the target species with no visualization of secondary peaks with lower melting temperature (primer dimers).

The standard curve method was used to determine the qPCR reaction efficiency and the limit of detection (LOD), which was defined as the lowest amount of target DNA in a sample that can be reliably detected with > 95% amplification success [57]. Standard curves were constructed using 17 ng of gDNA from *A. purpuratus* that was obtained using a commercial DNA extraction kit based on magnetic beads technology (iPrep TM purification instrument, Invitrogen, Carlsbad, CA, USA). The gDNA was 10-fold serially diluted from 17 ng to 1.7 × 10^−8^ ng, and subjected to qPCR to construct the standard curves, using 2 µL of each standard and three technical replicates for each dilution. qPCR was performed using the qPCR mix reactions and cycling protocols shown in panel A of S1 Fig, with the only modification being that total amplification cycles were 45 instead of 40. qPCR efficiency was calculated by plotting the Ct values of the dilution series against the logarithm of the DNA concentration from each standard dilution. The quantification cycles (Ct values) and amplification efficiency were calculated using the LightCycler 480 II Software (version 1.5.1.62) under the “Abs Quant/2nd Derivative Max” analyses, from the slope of the linear regression using the equation E = 10^(− 1/slope)^. Mean values, standard deviations, standard curve figures, and *R*^*2*^ values were obtained with Excel 2016 for macOS.

### 2.6. Direct standard PCR and qPCR assays for the authentication of commercial scallop samples

To further evaluate the performance of the *A. purpuratus* SSP set ARGOF/ARPU129R as a potential field deployable tool, we performed a direct qPCR assay (without DNA isolation step) using genetic material collected by non-invasive sampling of 5 fresh and 5 frozen individuals of *A. purpuratus*, bought in a local market and a supermarket, respectively. The non-invasive sampling was performed by gently rubbing a sterile cotton swab against the scallops’ adductor muscles (panel A in S2 Fig). Swabs used in fresh and frozen scallops were immediately placed in 1.5 mL Eppendorf tubes containing 1400 µL and 400 µL of 1 × phosphate buffer saline (PBS), respectively. Before standard PCR and qPCR amplifications, the Eppendorf tubes were vortexed, spun down, and incubated for 10 min at 55 °C. Direct standard PCR and qPCR reactions were performed following the same PCR and qPCR amplification protocols described in panel A of S1 Fig.

### 2.7. *In-situ* validation assays of species-specific primers by qPCR in eDNA samples

The reliability and specificity of the SSP sets targeting *A. purpuratus* were further tested to ascertain its presence or absence in field environmental samples. qPCR amplifications were conducted in triplicate in a LightCycler 480 II (Roche Diagnostics GmbH, Penzberg, Germany), the 2x SYBR Green I Master (Roche Diagnostics, Mannheim, Germany) kit was used in all qPCR eDNA runs on 96-well reaction plates (Roche Diagnostics GmbH, Mannheim, Germany) with the parameters shown in panel A of S1 Fig, with two main modifications: 1 µL of each primer (10 µM) was used making a final SSP concentration of 0.5 µM in each reaction and 45 amplification cycles were used instead of 40. Environmental DNA amplifications were considered as positive detections only if at least two out of three qPCR technical replicates displayed a fluorescent signal above the threshold in one field sample [58] and showed 100% sequence identity with the target species after Sanger sequencing.

To quantify the DNA concentration present in each environmental sample, a standard curve was constructed using gDNA from *A. purpuratus* extracted with magnetic beads technology (iPrep TM purification instrument, Invitrogen, Carlsbad, CA, USA) in 10-fold serial dilutions ranging from 10 ng to 1.0 × 10^-11^ ng. qPCR conditions were the same as described in the previous paragraph. The standard curve method was also used to determine the limit of detection (LOD), which was defined as the lowest concentration of the standard dilutions that gave at least one positive amplification out of the 3 replicates. This LOD definition is in accord with previous eDNA published articles based on species-specific assays of different aquatic organisms [59–61].

#### 2.7.1. Inhibition testing.

To determine the presence of inhibitors in the environmental samples that can lead to potential false negative results, all eDNA samples and negative field controls were spiked with an exogenous internal positive control (IPC) obtained from a freshwater fish species, ensuring the complete absence of the exogenous DNA control in the marine environmental samples. The IPC consisted of 1 µL of 5 ng/µL of gDNA of a male specimen of the Amazonian giant fish *Arapaima gigas* (*paiche*) that was qPCR-amplified using the primer set MSR_129 (International Patent WO 2024/005656 A1) previously developed for the specific genotypic sexing of the *paiche* [62]. eDNA samples were deemed inhibited if qPCR resulted in a Ct difference > 2 between the eDNA samples and the positive control of pure *paiche* DNA [25]. The IPC qPCR assay was performed in duplicate using 10 µL of 2x SYBR Green I Master (Roche Diagnostics, Mannheim, Germany), 0.2 µL each MSR_129 primer, 1 µL of *paiche* gDNA (5 ng/µL), and either 2 µL of eDNA template or 2 µL ultrapure H_2_O for the no-template control, in a 20 µL total reaction volume. The qPCR amplification parameters were identical to those described by López-Landavery et al. [62].

#### 2.7.2. Authentication of positive eDNA amplicons by Sanger sequencing.

In order to verify the species identity of the positive eDNA amplifications, positive qPCR amplicons (one per each positive replicate) were Sanger sequenced bidirectionally using the same SSP set ARGOF/ARPU129R. Forward and reverse electropherograms were trimmed and aligned to obtain consensus sequences that were contrasted against the complete mitochondrial genome of *A. purpuratus* (GenBank accession KF601246).

### 2.8. Applicability of species-specific primers for the authentication of cooked fish samples from restaurants

To assess the effectiveness of our SSP sets for authenticating commercially cooked samples collected from restaurants, we tested various seafood presentations, including ceviche, stewed, fried, seafood rice, and mixed fried seafood. The collected samples included different types of seafood such as flounders, grunts, grape-eye seabasses, rock seabasses, scallops, and squids. We conducted standard PCR-SSP assays to authenticate cooked samples labeled as scallop, squid, and flounder. For identifying samples labeled as rock seabass and grape-eye seabass, we performed two different duplex PCRs. The first duplex PCR for “*cabrillas*” (rock seabasses) utilized the SSP sets PACA163F/R, targeting *Paralabrax callaensis*, and PAHU288F/R, targeting *P. humeralis*, in a single reaction tube. Interpretation of results was based on the visualization of a single PCR product, either 163 bp or 288 bp in length. These fragment sizes indicated the presence of DNA from *P. callaensis* or *P. humeralis*, respectively. The species identity of the PCR products from cooked “*cabrilla*” samples was confirmed through bidirectional Sanger sequencing using the same SSPs.

For samples labeled as “*ojo de uva*”, which could refer to either the grape-eye seabass (*Hemilutjanus macrophthalmos*) or the mocosa ruff (*S. haedrichi*), we performed a duplex PCR with the SSP sets HEMA122F/R and SCHA244F/R. This allowed the accurate identification of both species in a single PCR reaction, given the distinct amplicon sizes of 122 bp for *H. macrophthalmos* and 244 bp for *S. haedrichi*. All PCR results were verified by 1.5% agarose gel (EMD Millipore, Billerica, MA, USA) electrophoresis. The species identities were verified through DNA sequencing analysis.

## 3. Results

During the evaluation of various putative SSPs in different shellfish and fish species, successful SSP assays were optimized and validated with 201 specimens from 10 target species (see Table 2). These species are naturally distributed from the Central to the South East Pacific and the South West Atlantic. All assays demonstrated high specificity, showing no nonspecific or cross-species amplifications.

**Table 2:** Species-specific primer sequences designed for the identification of 10 Peruvian marine species. Nucleotides written in lowercase characters represent intentionally introduced GC tail at the 5’ end of the species-specific primer. Ann T °C: annealing temperature.

Group	Species scientific name	Common name English/Spanish	Primer name	Sequence (5’-3’)	Sense	Ann T °C	Size (bp)	Gene
Bivalves	*Argopecten purpuratus*	Peruvian scallop/ Concha de abanico	ARGOF	CTTCTGTCTCTAGCTTGTTTTAGTG	Forward	61	129	16S
			ARPU129R	GCTAAGGGAAGTAACCTTCTAC	Reverse			
Cephalopods	*Doryteuthis gahi*	Patagonian longfin squid/ Calamar común	DOGA136F	ggCGATGAGAAGGTTTATT	Forward	60	136	COI
			DOGA136R	ccAAAGTTTCGATCGGTTAGTAA	Reverse			
Flounders	*Paralichthys adspersus*	Fine flounder/ Lenguado fino	PAAD165F	ATACCAAGTCCCCCTATTTATC	Forward	60	165	COI
			PAAD165R	TGTTGGTAGAGGATGGGATCA	Reverse			
	*Etropus ectenes*	Sole flounder/ Lenguado boca chica	ETROP162F	TATCAACATGAAGCCCACAT	Forward	60	162	COI
			ETROP162R	GGGTCAAAGAAGGTTGTGTTCAAA	Reverse			
Rock seabasses	*Paralabrax callaensis*	Southern seabass/ Cabrilla fina	PACA163F	ACCCTATGAAGCTTTAGACACCAGA	Forward	61	163	16S
			PACA163R	GCTCTGGGTTGTAAGAGAGTAAAA	Reverse			
	*Paralabrax humeralis*	Peruvian rock seabass/ Cabrilla común	PAHU288F	CGCAATCACTTGTCCC	Forward	62	288	16S
			PAHU288R	CTCTGGGTTGTAAGAGAGTTAAT	Reverse			
Seabass	*Hemilutjanus macrophthalmos*	Grape-eye seabass/ Ojo de uva	HEMA122F	AATCCTCGGGGCCATTAACTTCATC	Forward	60	122	COI
			HEMA122R	AAAGTAGAAGAAGAACGGCAGTG	Reverse			
Cojinovas	*Schedophilus haedrichi*	Mocosa ruff/ Cojinova mocosa	SCHA244F	AACTGGTTGAACAGTATATCCTCCT	Forward	60	244	COI
			SCHA244R	AGCAGCTAGGACAGGGAGAGATAAT	Reverse			
Groupers	*Alphestes immaculatus*	Pacific mutton hamlet/ Mero rojo	ALIM135F	gcGACACTAAAGCAGATCATAT	Forward	60	135	16S
			ALIM135R	GTAGTACATTCGGTCCTTAT	Reverse			
Grunts	*Anisotremus interruptus*	Burrito grunt/ Chita dorada	ANIN246F	TAGCTCACGCCGGAGCATCTGTC	Forward	64	246	COI
			ANIN246R	TGGTATTTAGATTTCGGTCCGTGAGA	Reverse			

**Fig 2:**
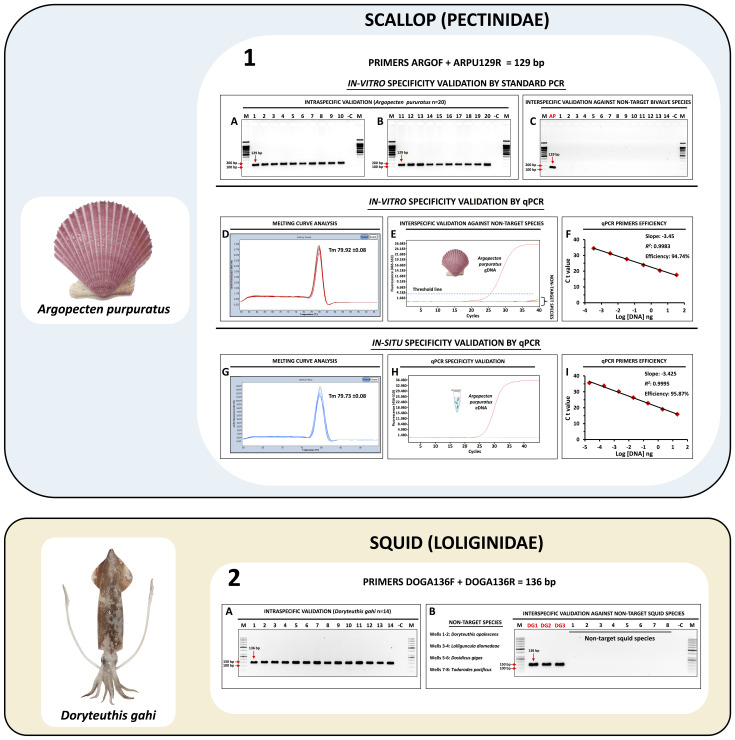
Specificity validation assay results for the species-specific primer targeting *A. purpuratus* (*in-vitro* and *in-situ*) and *D. gahi* (*in-vitro*). Panel 1A and 1B: Intraspecific validation of the primer set ARGOF/ARPU129R in 20 individuals of *A. purpuratus* (amplicon size 129 bp). Panel 1C: Interspecific validation results of ARGOF/ARPU129R against non-target bivalve species, well “AP” is the positive control (*A. purpuratus*), wells 1 to 8: *A. ventricosus*, well 9: *Pteria sterna*, well 10: *Striostrea prismatica*, well 11: *Atrina maura*, well 12: *Perumytilus purpuratus*, well 13: *Aulacomya atra*, well 14: *Gari solida*. *In-vitro* specificity validation by qPCR analysis of fresh individuals of *A. purpuratus* displayed in panel 1D: melting curve analysis result, panel 1E: specificity validation against non-target bivalve species (*A. maura*, *A. atra*, *A. ventricosus*, *G. solida*, *P. purpuratus*, *P. sterna*, and *S. prismatica*), panel 1F: qPCR efficiency results obtained by the standard curve method. *In-situ* specificity validation by qPCR analysis of *A. purpuratus* eDNA displayed in panel 1G: melting curve analysis result, panel 1H: specific detection of *A. purpuratus* in eDNA samples, panel I: eDNA-qPCR efficiency results obtained by the standard curve method. Panel 2A: intraspecific validation results of the primer set DOGA136F/R in 14 individuals of *D. gahi* (amplicon size 136 bp). Panel 2B: interspecific validation results of DOGA136F/R against non-target squid species, wells “DG1” to “DG3” are positive controls for *D. gahi*.

**Fig 3:**
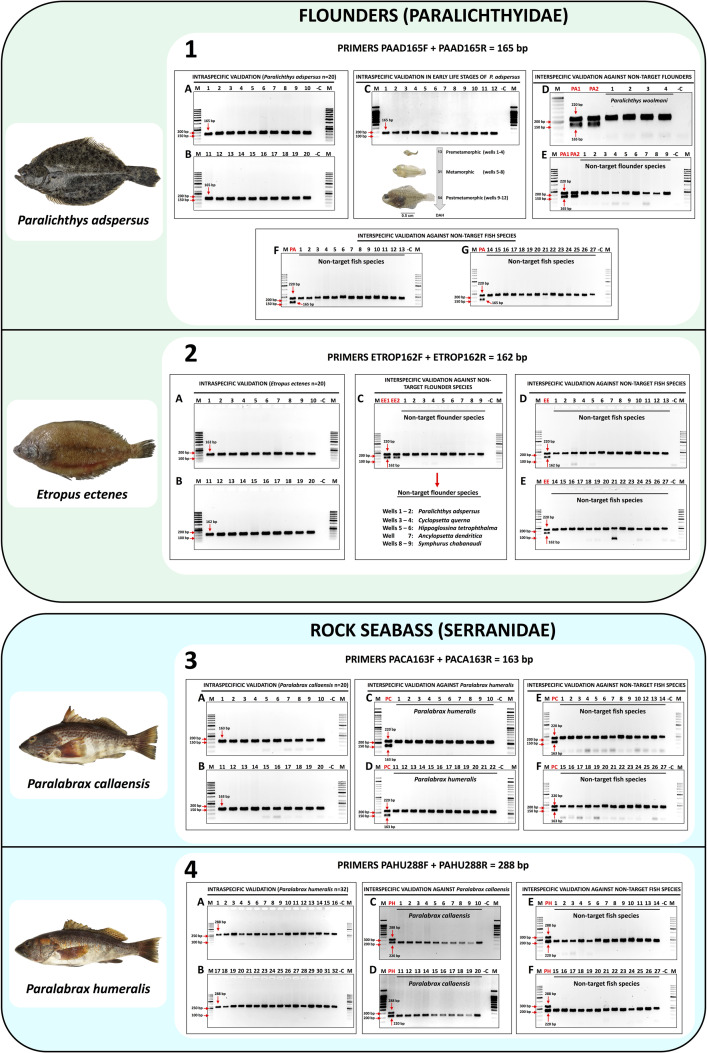
Standardized *in-vitro* specificity validation assay results for the species-specific primers targeting (1) *Paralichthys adspersus*, (2) *Etropus ectenes*, (3) *Paralabrax callaensis*, and (4) *P. humeralis.* Panels 1A and 1B: intraspecific PCR validation results of the primer set PAAD165F/R in 20 individuals of *P. adspersus* (amplicon size 165 bp). Panel 1C: Intraspecific PCR validation results of the primer set PAAD165F/R in early life stages of *P. adspersus*. Panels 1D and 1E: interspecific PCR validation results of PAAD165F/R against non-target flounders, wells “PA” are positive controls of *P. adspersus*. Panels 1F and 1G: interspecific PCR validation of PAAD165F/R against non-target fish species listed in S1 Table. Panels 2A and 2B: intraspecific PCR validation results of primer set ETROP162F/R in 20 individuals of *E. ectenes*. Panel 2C: interspecific PCR validation results of ETROP162F/R against non-target flounders, wells “EE” are positive controls of *E. ectenes*. Panels 2D and 2E: interspecific PCR validation results of ETROP162F/R against non-target fish species listed in S1 Table. Panels 3A and 3B: intraspecific PCR validation results of primer set PACA163F/R in 20 individuals of *P. callaensis*. Panels 3C and 3D: interspecific PCR validation results of PACA163F/R against the congeneric *P. humeralis*, wells “PC” are positive controls of *P. callaensis*. Panels 3E and 3F: interspecific PCR validation results of PACA163F/R against non-target fish species listed in S1 Table. Panels 4A and 4B: intraspecific PCR validation results of primer set PAHU288F/R in 32 individuals of *P. humeralis*. Panels 4C and 4D: interspecific PCR validation results of PAHU288F/R against the congeneric *P. callaensis*, wells “PH” are positive controls of *P. humeralis*. Panels 4E and 4F: interspecific PCR validation results of PAHU288F/R against non-target fish species listed in S1 Table.

**Fig 4:**
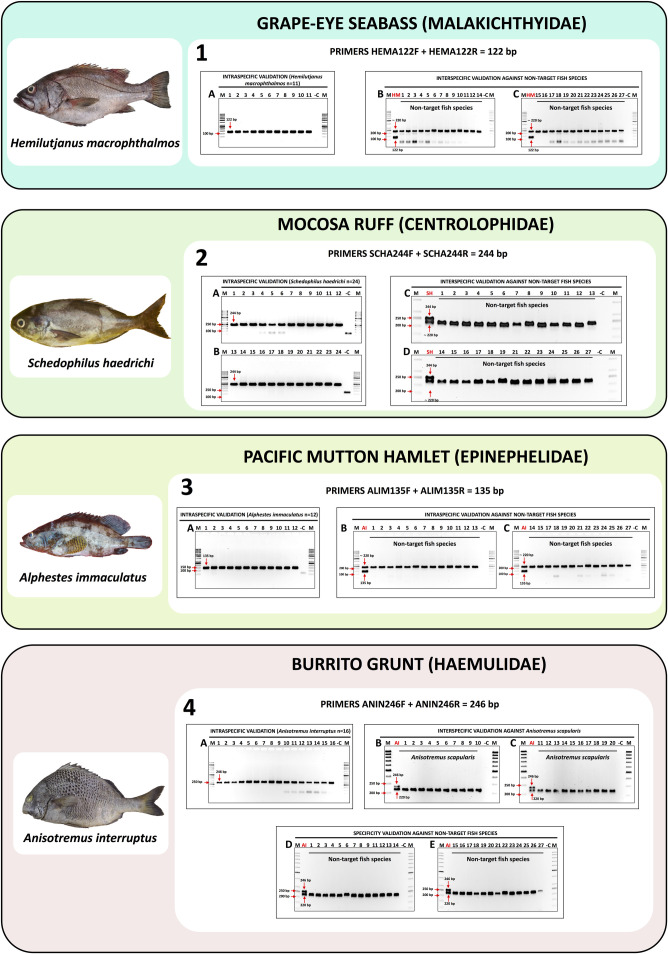
Standardized *in-vitro* specificity validation assay results for the species-specific primers targeting (1) *Hemilutjanus macrophthalmos*, (2) *Schedophilus haedrichi*, (3) *Alphestes immaculatus*, and (4) *Anisotremus interruptus.* Panel 1A: intraspecific PCR validation results of primer set HEMA122F/R in 11 individuals of *H. macrophthalmos*. Panels 1B and 1C: interspecific PCR validation results of HEMA122F/R against non-target fish species listed in S1 Table, wells “HM” are the positive controls of *H. macrophthalmos*. Panel 2A and 2B: intraspecific PCR validation results of primer set SCHA244F/R in 24 individuals of *S. haedrichi*. Panels 2C and 2D: interspecific PCR validation results of SCHA244F/R against non-target fish species listed in S1 Table, wells “SH” are the positive controls of *S. haedrichi*. Panel 3A: intraspecific PCR validation results of primer set ALIM135F/R in 12 individuals of *A. immaculatus*. Panels 3B and 3C: interspecific PCR validation results of ALIM135F/R against non-target fish species listed in S1 Table, wells “AI” are the positive control of *A. immaculatus*. Panel 4A: intraspecific PCR validation results of primer set ANIN246F/R in 16 individuals of *A. interruptus*. Panels 4B and 4C: interspecific PCR validation results of primer set ANIN246F/R against congeneric *A. scapularis*, wells “AI” are the positive control of *A. interruptus*. Panels 4D and 4E: interspecific PCR validation results of ANIN246F/R against non-target fish species listed in S1 Table.

The shellfish species group that has validated molecular identification assays includes the Peruvian scallop *A. purpuratus* (panel 1 in Fig 2) and the Patagonian longfin squid *D. gahi* (panel 2 in Fig 2). The fish species group consists of the fine flounder *P. adspersus* (panel 1 in Fig 3) and the sole flounder *Etropus ectenes* (panel 2 in Fig 3), the southern rock bass *P. callaensis* (panel 3 in Fig 3) and the Peruvian rock seabass *P. humeralis* (panel 4 in Fig 3), the grape-eye seabass *H. macrophthalmos* (panel 1 in Fig 4), the mocosa ruff *S. haedrichi* (panel 2 in Fig 4), the Pacific mutton hamlet *Alphestes immaculatus* (panel 3 in Fig 4), and the burrito grunt *Anisotremus interruptus* (panel 4 in Fig 4).

Our primer standardization results showed that the 16S rRNA gene was the best candidate for SSPs design in *A. immaculatus*, *A. purpuratus*, *P. callaensis*, and *P. humeralis*, whereas the COI gene gave best results in *A. interruptus*, *D. hagi*, *E. ectenes*, *H. macrophthalmos*, *P. adspersus*, and *S. haedrichi*. All novel SSP sequences, optimal annealing temperatures, and expected amplicon sizes are shown in Table 2. PCR mix protocols and amplification conditions used during the in *in-vitro* validation testing of each SSP are shown in panels A to J from S1 Fig.

The SSP set designed for the identification of *A. purpuratus* was evaluated through three stages including *in-silico*, *in-vitro* (standard and qPCR using gDNA from target and non-target scallop tissues), and *in-situ* (qPCR using eDNA from seawater); while the SSPs targeting squid and fish species were validated through *in-silico* and *in-vitro* stages. The annealing temperature of the SSPs ranged from 60 to 64 °C, ensuring a high stringency of the PCR and qPCR assays. Targeted PCR products ranged from 129 to 288 bp, which enabled the successful amplification of degraded DNA commonly present in eDNA samples and processed seafood. A total of 164 DNA sequences belonging to 28 seafood species were generated in this study (S3 Table).

### 3.1. *In-silico* specificity validation assays of species-specific primers

The *in-silico* specificity validation assays of our novel SSPs are depicted in S3 Fig to S5 Fig. All analyzed DNA sequences from target species used in the *in-silico* intraspecific and interspecific validation analyses are presented in Supplementary FASTA files S1 File to S13 File. These ranged from 4 sequences in *E. ectenes* to 175 sequences in *A. interruptus*. The *in-silico* validation assay tests against non-target species showed the presence from one to several mismatches positioned along the primer hybridization regions including the 3’ end, which is known to result in the most detrimental effects for polymerase activity [11,12]. The results of the *in-silico* analysis of the SSP set for *A. purpuratus* can be found in detail in S3 Fig. A “GC” tail was deliberately introduced at the 5’ end of the SSPs for *D. gahi* and the forward SSP for *A. immaculatus* (see Table 2) to meet the minimum percentage of recommended GC content (40%) and to ensure a more stable primer/template binding [63] during PCR amplification.

The *in-silico* validation assays using the Primer–BLAST tool demonstrated the high species-specificity of each SSP. These results also indicated that the SSP set targeting *D. gahi* (DOGA136F/R) would amplify fragments of 794 and 2546 bp in the diamond squid *Thysanoteuthis rhombus* (Thysanoteuthidae). However, this elusive cosmopolitan squid species is only targeted in Japanese waters [64], while in Peru it has no commercial value and only few incidents were reported as bycatch [65]. The Primer–BLAST results of the SSP primer set PAHU288F/R targeting *P. humeralis* showed a single mismatch in the hybridization region of the reverse primer of its congeneric relative *P. nebulifer*, suggesting a possible PCR cross-amplification in that species. However, *P. nebulifer* only occurs in the Northern Eastern Pacific from Santa Cruz in central California (USA) to Magdalena Bay in Baja California (Mexico) [66]. The primer set ETROP162F/R was originally designed based on the mitochondrial COI sequence of *E. crossotus*, due to the lack of reference sequences of *E. ectenes* at the time of primer design. Therefore, the Primer–BLAST analysis of the SSP set ETROP162F/R indicated that it could be amplified in both congeneric species that co-occur in northern Peru [41]. We must highlight however that catches of *E. ectenes* are significantly higher than those of *E. crossotus* and only landings of the former species are reported to the species level in official landing records [67–69].

### 3.2. *In-vitro* and *in-situ* validation of species-specific primers in *Argopecten purpuratus*

#### 3.2.1. *In-vitro* specificity validation of species-specific primers using standard PCR and qPCR.

In a first *in-vitro* validation test by standard PCR, the SSP set for *A. purpuratus* (ARGOF/ARPU129R) amplified a single specific PCR product of 129 bp (electrophoresis gels A and B from panel 1 in Fig 2), without primer dimer formation, in all tested individuals (n = 20) collected from two distant bays located in Piura and Pisco regions (Table 1). The results of the specificity PCR validation test in non-target species are shown in the electrophoresis gel C from panel 1 in Fig 2. No cross-species reactions were observed at any of the 7 bivalve species that included DNA of a congeneric scallop (*A*. *ventricosus*), two oysters (*Pteria sterna* and *Striostrea prismatica*), a penshell (*Atrina maura*), a clam (*Gari solida*), and two mussel species (*Aulacomya atra* and *Perumytilus purpuratus*).

The high specificity of the SSP set ARGOF/ARPU129R was also confirmed in a second *in-vitro* validation by quantitative PCR results. A melting curve analysis of the qPCR products showed a single sharp peak with an average melting temperature (Tm) value of 79.79 °C ± 0.1 in all analyzed *A. purpuratus* samples (panel 1D in Fig 2), indicating the specific yield of a single qPCR product without primer dimer formation or non-specific products. qPCR specificity validation against non-target bivalve species resulted in no amplification curve formation during the 45 cycles (panel 1E in Fig 2), confirming the absence of cross-species amplification.

Based on the results of the standard curve analysis (panel 1F in Fig 2), the LOD of the qPCR for *A. purpuratus* was 3.94 × 10^−4^ ng in 2 µL of DNA template (mean Ct 34.55). A qPCR efficiency of 94.74% was obtained, which is within the acceptable range of highly efficient primers (90–110%) as described in the MIQE Guidelines [57].

**Authentication of commercial *Argopecten purpuratus* samples using direct standard PCR and qPCR:** A schematic representation of the direct qPCR and standard PCR detection assay for *A. purpuratus* is depicted in S2 Fig. The swabbing, preservation, and DNA elution steps lasted just 12 min. All fresh and processed samples were successfully identified by our direct qPCR (panel B in S2 Fig) and standard PCR (panel C in S2 Fig) assays. The direct qPCR assay displayed positive identification signals between 50–60 min (Ct values ranged from 19.55 to 26.86). The results of direct standard PCR assay, which were visualized in an agarose gel electrophoresis (panel C in S2 Fig), were obtained in a total time of 150 min.

#### 3.2.2. *In-situ* specificity validation of species-specific primers targeting *Argopecten purpuratus* in eDNA samples using qPCR and Sanger sequencing.

The *in-situ* qPCR validation assay targeting *A. purpuratus* in eDNA samples showed that all positive controls (tissue-derived DNA, run in triplicate) included in each qPCR run were successfully amplified with Ct values corresponding to its DNA concentration, whereas the negative control replicates (qPCR water as DNA template and filtered distilled water from eDNA field surveys) gave no fluorescence signal, validating the qPCR assays. Validation of the positive qPCR products using Sanger sequencing confirmed that all eDNA amplicons belonged to *A. purpuratus* (GenBank accessions from PP087144 to PP087154, 100% identity match with *A. purpuratus* mitogenome reference sequence KF601246). Environmental DNA of the Peruvian scallop was detected in all replicates (3/3) from all sampling stations in Sechura Bay (map B in Fig 1), Samanco Bay (map C in Fig 1), and Tortugas Bay (map D in Fig 1), except for La Cruz offshore station in Tumbes region (map A in Fig 1), which is out of the natural distribution range of *A. purpuratus* (Peña, 2001). eDNA concentrations ranged from 2.69 × 10^−1^ ng (Parachique station) to 8.52 × 10^−4^ ng (Tortugas 5 station). The Parachique station in Sechura Bay was the only location from which eDNA samples were collected at three different layers of the water column (bottom, midwater, and surface) and where the presence of the Peruvian scallop was confirmed by visual inspection during bottom water sampling. *A. purpuratus* eDNA concentrations from that station were similar among bottom (1.68 × 10^−2^ ng, Ct 26.53 ± 0.11), middle (2.69 × 10^−1^ ng, Ct 24.3 ± 0.03), and surface (2.0 × 10^−1^ ng, Ct 24.75 ± 0.06) levels. eDNA extractions from Tortugas 1 and Tortugas 3 obtained from half filters generated lower *A. purpuratus* eDNA amounts than those extractions using quarter filters (Table 3), possibly due to the presence of inhibitors in those samples.

**Table 3:** eDNA identification results of the species-specific primers targeting *A. purpuratus*. Information from each sampling location, collection dates and season, geographic coordinates, filtered water volume and level of the water column (B: bottom, M: midwater, S: surface), and qPCR results are shown. Sampling stations with an asterisk (*) denote eDNA samples extracted from a quarter filter. The “+” and “−“ signs indicate positive and negative detections, respectively.

Location	Site information	Sampling station	Geographic coordinates	Sampling date (Season)	Vol. (L)	Level	*A. purpuratus* (GenBank accession)	qPCR Ct	Concentration (ng)
Sechura Bay (Piura region)	Open bay with wild populations of *A. purpuratus* and *A. ventricosus*. This bay is also used for *A. purpuratus* farming (mainly sea ranching, hanging lantern nets to a lesser extent)	Parachique	05°45’9.7“S, 080°53’52.6”W	October 14, 2017 (Spring)	0.5	B	+ (PP087144)	26.53 ± 0.11	1.68 × 10^−2^ ± 1.13 × 10^−3^
					0.5	M	+ (PP087145)	24.3 ± 0.03	2.69 × 10^−1^ ± 5.55 × 10^−3^
					0.5	S	+ (PP087146)	24.75 ± 0.06	2.0 × 10^−1^ ± 7.36 × 10^−3^
		Bayóvar	05°48’25.8“S, 081°00’34.9”W	May 11, 2022 (Autumn)	1	B	+ (PP087147)	28.42 ± 0.11	2.13 × 10^−2^ ± 1.47 × 10^−3^
		Matacaballo	5°38’10.2“S 80°51’40.7”W	May 12, 2022 (Autumn)	1		+ (PP087148)	32.67 ± 0.08	1.60 × 10^−3^ ± 7.99 × 10^−5^
Samanco Bay (Ancash region)	Semi-enclosed bay with wild and cultured populations (hanging lantern nets) of *A. purpuratus*	El Dorado	09°12’24“S, 078°31’46”W	February 12, 2019 (Summer)	1	S	+ (PP087149)	29.26 ± 0.21	6.41 × 10^−3^ ± 8.19 × 10^−4^
Tortugas Bay (Ancash region)	Semi-enclosed bay with wild populations of *A. purpuratus*	Tortugas 1	09°22’06.8“S, 078°25’03.3”W	April 29, 2022 (Autumn)	1	B	+ (PP087150)	32.14 ± 0.14	1.36 × 10^−3^ ± 1.13 × 10^−4^
		Tortugas 1*					+ (PP087151)	27.75 ± 0.08	7.74 × 10^−3^ ± 3.89 × 10^−4^
		Tortugas 2	09°21’38.2“S, 078°25’00.6”W		1		+ (PP087152)	32.94 ± 0.26	8.52 × 10^−4^ ± 1.36 × 10^−4^
		Tortugas 3	09°21’42.5“S, 078°25’10.4”W		1		+ (PP087153)	31.28 ± 0.24	1.86 × 10^−3^ ± 2.60 × 10^−4^
		Tortugas 3*					+ (PP087154)	29.34 ± 0.22	2.88 × 10^−3^ ± 3.75 × 10^−4^
La Cruz (Tumbes Region)	Offshore sampling station without historical records of *A. purpuratus*	La Cruz	3°34’35.1’‘S, 80°37’3.3’‘W	December 12, 2017 (Spring)	1	S	—	—	—

**Inhibition test and LOD:** The eDNA inhibition test indicated that all blank control samples obtained in all sampling stations and the eDNA samples collected at the Parachique station (Sechura Bay) showed a Ct shift < 2 compared to that of the positive exogenous DNA control (*A. gigas*), suggesting the absence of inhibitory substances. All the remaining eDNA samples displayed a Ct shift > 2 and therefore were deemed inhibited. In an attempt to overcome this issue, inhibited samples were diluted (1:1, 1:5, 1:10, and 1:20) and qPCR amplified using 0.5 µL of bovine serum albumin (BSA, 20 mg/ml), resulting in positive detections of the target species in all inhibited samples. The LOD of our *in-situ* eDNA assay for *A. purpuratus* was 2 × 10^−5^ ng in 2 µL of eDNA template (Ct 35.62), with a qPCR efficiency of 95.87% (panel I in Fig 2).

### 3.3. *In-vitro* specificity validation of species-specific primers using standard PCR in squid samples

The *in-vitro* specificity validation results demonstrated the high performance of the SSP set DOGA136F/R targeting the Patagonian squid *D. gahi*, as shown in electrophoresis gel A in panel 2 from Fig 2, where all tested target individuals (n = 14, 100%) were successfully amplified. Electrophoresis gel B in panel 2 from Fig 2 demonstrated that no cross-species reactions occurred when the SSP was tested against 4 non-target squid species including two economically important species from Peru: *Lolliguncula diomedeae* and *Dosidicus gigas*.

### 3.4. *In-vitro* specificity validation of species-specific primers using standard PCR in fish samples

The *in-vitro* specificity validation assays were evaluated using from 11 to 32 specimens of the target fish species, resulting in the specific amplification of a single specific amplicon in all tested individuals. Thus, species-specific PCR products of the following sizes were obtained for each target species: 165 bp in 32 specimens of *P. adspersus* corresponding to 20 adults and 12 larvae/juveniles (electrophoresis gels A to C in panel 1 from Fig 3), 162 bp in 20 specimens of *E. ectenes* (electrophoresis gels A and B in panel 2 from Fig 3), 163 bp in 20 specimens of *P. callaensis* (electrophoresis gels A and B in panel 3 from Fig 3), 288 bp in 32 specimens of *P. humeralis* (electrophoresis gels A and B in panel 4 from Fig 3), 122 bp in 11 specimens of *H. macrophthalmos* (electrophoresis gel A in panel 1 from Fig 4), 244 bp in 24 specimens of *S. haedrichi* (electrophoresis gels A and B in panel 2 from Fig 4), 135 bp in 12 specimens of *A. immaculatus* (electrophoresis gel A in panel 3 from Fig 4), and 246 bp in 16 specimens of *A. interruptus* (electrophoresis gels A in panel 4 from Fig 4).

The results from the duplex PCR *in-vitro* specificity assays against non-target fish species demonstrated the high specificity of all the SSPs developed herein. All electrophoresis gels showing the results from the duplex PCR *in-vitro* specificity assays against non-target fish species are shown in Fig 3 and Fig 4. All non-target fish species (listed in S1 Table) amplified only the endogenous control band of about 220 bp belonging to the 12S rRNA gene, while only the positive control samples (DNA from target species) yielded two bands, corresponding to the species-specific amplicon and the endogenous control band. Furthermore, specificity assays against non-target congeneric species were evaluated using the SSPs targeting *Paralichthys adspersus*, *Paralabrax callaensis*, *Paralabrax humeralis*, and *Anisotremus interruptus*, using DNA from congeneric *Paralichthys woolmani* (n = 4, electrophoresis gel D in panel 1 from Fig 3), *P. humeralis* (n = 22, electrophoresis gels C and D in panel 3 from Fig 3), *P. callaensis* (n = 20, electrophoresis gels C and D in panel 4 from Fig 3), and *Anisotremus scapularis* (n = 20, electrophoresis gels B and C in panel 4 from Fig 4), respectively. No cross-reactions were observed at any of the DNA samples belonging to the congeneric species.

### 3.5. Application of species-specific primers for the authentication of cooked seafood samples from restaurants

All restaurant samples identified at the species-level using our PCR-SSP assays are listed in Table 4. We successfully identified 11 cooked samples with our novel PCR-SPS assays, achieving 100% identification success. These included five samples labeled as scallop (*A. purpuratus*, 129 bp, n = 1), squid (*D. gahi*, 136 bp, n = 3), and burrito grunt (*A. interruptus*, 246 bp, n = 1). Identification was performed using the standard PCR-SSP amplification protocols and master mix reactions detailed in S1 Fig (panel A: *A. purpuratus*, panel B: *D. gahi*, and panel I: *A. interruptus*). Further DNA sequencing analyses of each PCR product from these samples confirmed our identification results based on the SSPs. The DNA sequences have been deposited in the GenBank database and their accessions are shown in Table 4.

**Table 4:** Authentication of cooked seafood samples collected from Peruvian restaurants.

Code	Sample label Spanish (English)	Date	Presentation	Locality (Region)	Species-Specific Primer set	Amplicon size (bp)	Rapid molecular diagnosis	Species identity by DNA sequencing (GenBank accession)
**1**	Cabrilla (rock seabass)	May 7, 2019	Fried	Piura (Piura)	**PACA163F/R **+ PAHU288F/R	163	*Paralabrax callaensis*	*P. callaensis* (PP087227)
**2**	Cabrillon (jumbo rock seabass)	May 7, 2019	Stewed	Piura (Piura)	**PACA163F/R **+ PAHU288F/R	163	*Paralabrax callaensis*	*P. callaensis* (PP087228)
**3**	Cabrilla (rock seabass)	May 7, 2019	Ceviche	Chimbote (Ancash)	PACA163F/R + **PAHU288F/R**	288	*Paralabrax humeralis*	*P. humeralis* (PP087229)
**4**	Cabrilla (rock seabass)	May 7, 2019	Stewed	Chimbote (Ancash)	PACA163F/R + **PAHU288F/R**	288	*Paralabrax humeralis*	*P. humeralis* (PV491563)
**5**	Calamar (squid)	May 7, 2019	Seafood rice	Piura (Piura)	DOGA136F/R	136	*Doryteuthis gahi*	*Doryteuthis gahi* (see S3 Table)**
**6**	Berrugata	May 10, 2019	Fried	Piura (Piura)	ANIN246F/R	246	*Anisotremus interruptus*	*A. interruptus* (PV562156)
**7**	Ojo de uva (grape-eye seabass)	May 11, 2019	Ceviche	Huanchaco (La Libertad)	SCHA244F/R* + HEMA122F/R	244	*Schedophilus haedrichi*	*S. haedrichi* (PV562157)
**8**	Concha de abanico (Peruvian scallop)	March 24, 2024	Seafood rice	Chimbote (Ancash)	ARGOF + ARPU129R	129	*Argopecten purpuratus*	*A. purpuratus* (PV491562)
**9**	Ojo de uva (grape-eye seabass)	April 22, 2024	Stewed	Tumbes (Tumbes)	SCHA244F/R** **+ HEMA122F/R*	122	*Hemilutjanus macrophthalmos*	*H. macrophthalmos* (see S3 Table)**
**10**	Calamar (squid)	April 24, 2024	Ceviche	Chimbote (Ancash)	DOGA136F/R	136	*Doryteuthis gahi*	*Doryteuthis gahi* (see S3 Table)**
**11**	Calamar (squid)	April 24, 2024	Seafood rice	Chimbote (Ancash)	DOGA136F/R	136	*Doryteuthis gahi*	*Doryteuthis gahi* (see S3 Table)**
**12**	Calamar* (squid)	May 9, 2019	Seafood rice	Chiclayo (Lambayeque)	DOGA136F/R	--	--	*Dosidicus gigas* (PV562159)
**13**	Calamar* (squid)	May 9, 2019	Mix fried seafood	Chiclayo (Lambayeque)	DOGA136F/R	--	--	*Dosidicus gigas* (PV562160)
**14**	Lenguado* (flounder)	March 24, 2024	Fried fillet	Zorritos (Tumbes)	PAAD165F/R ETROP162F/R	--	--	*Cyclopsetta querna* (PV505011, PV562161)
**15**	Lenguado* (flounder)	April 22, 2024	Fried fillet	Tumbes (Tumbes)	PAAD165F/R ETROP162F/R	--	--	*Paralichthys woolmani* (PV505012)

*These samples did not contain DNA from the squid and flounder species targeted by our SSPs, their identification to the species level was achieved by DNA barcoding approach.

**These sequences are shown in S3 Table because the GenBank database no longer accepts DNA sequences shorter than 150 nucleotides.

**Fig 5:**
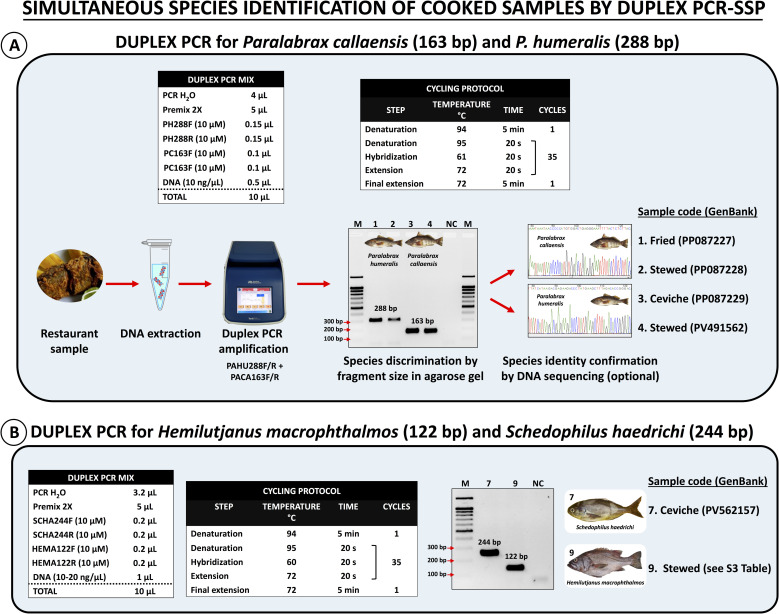
Duplex PCR-SSPs for the authentication of cooked seafood samples: panel A: identification protocol for *Paralabrax callaensis* and *P. humeralis*, and panel B: identification protocol for *Hemilutjanus macrophthalmos* and *Schedophilus haedrichi.*

Four additional fish species were identified in two independent duplex PCR-SSP assays. The amplification protocol and master mix reaction of the first duplex PCR-SSP assay are detailed in panel A of Fig 5. This assay included SSPs for the two congeneric species: *P. callaensis* (PACA163F/R) and *P. humeralis* (PAHU288F/R). It successfully identified four samples—one ceviche, one fried, and two stewed—based on the presence of species-specific bands of the expected sizes: 163 bp for *P. callaensis* and 288 bp for *P. humeralis* (see Panel A in Fig 5). The species identities of the PCR products from the cooked “*cabrilla*” samples were further verified by Sanger sequencing (GenBank accessions are listed in Table 4).

The amplification protocol and master mix reaction for the second duplex PCR-SSP assay are shown in panel B of Fig 5. This assay targeted two species commonly marketed as “*ojo de uva*” (Spanish for grape-eye): *H. macrophthalmos* (HEMA122F/R) and *S. haedrichi* (SCHA244F/R). It resulted in the simultaneous identification of both species in a stewed sample (*H. macrophthalmos* at 122 bp) and a ceviche sample (*S. haedrichi* at 244 bp) (refer to panel B in Fig 5). DNA sequencing analyses were also performed to confirm the species identification obtained with this second duplex PCR-SSP (GenBank accessions are listed in Table 4).

Four samples labeled as squid (codes 12 and 13 in Table 4) and flounder (codes 14 and 15 in Table 4) did not produce a PCR product when tested with our PCR-SSP assays designed to target the Patagonian squid and the fine flounder. Subsequent DNA barcoding analyses revealed that these samples actually belong to different species: the Humboldt squid (*D. gigas*) for samples code 12 and 13 (GenBank acesss PV562159 and PV562160), the toothed flounder (*C. querna*) for sample code14 (Genbank access PV505011), and the speckled flounder (*P. woolmani*) for sample code 15 (GenBank access PV505012).

## 4. Discussion

This study developed and validated various SSP detection assays targeting 10 commercially important fish and shellfish species from Peru. These assays were successfully evaluated in different scenarios including eDNA monitoring, early-life stage identification, and seafood products authentication. The novelty of this study lies not only in the wide range of genetic material analyzed—such as DNA from early life stages and adult individuals, forensic samples, commercially cooked and processed products, environmental DNA from seawater, and tissue swabs—but also in the diverse PCR techniques employed for molecular detection. These techniques included standard PCR, duplex PCR, qPCR, direct PCR and qPCR, eDNA qPCR, and DNA sequencing.

The Peruvian scallop, *A*. *purpuratus*, represents the most important scallop species along the Pacific coast of South America [70,71]. Its distribution extends from Paita in Peru (5° S) to Valparaíso in Chile (33° S) [72], where it is commercially exploited. Peruvian production is exported to at least 19 countries [73]. The SSP assay for *A. purpuratus* was validated via standard PCR and qPCR methods, demonstrating high specificity and efficiency in amplifying tissue-extracted DNA from both fresh and commercially processed samples, as well as swab samples, without the need for prior DNA isolation (direct PCR). No cross-species reactions were observed during the PCR and qPCR *in-vitro* validations against non-target bivalve species, thereby demonstrating its potential applicability for detecting mislabeling. The Peruvian scallop has been documented to be involved in substitution and mislabeling in different countries. For example, Parrondo et al. [74] identified cooked and frozen *A. purpuratus* samples incorrectly labeled as “*zamburiña*” (Spanish vernacular name for *Mimachlamys varia*) or substituted with “*volandeiras*” (*Aequipecten opercularis*) in Spanish restaurants and supermarkets. Similarly, Näumann et al. [75] and Klapper and Schröder [8] detected *A. purpuratus* mislabeled as *Pecten* spp. in commercial samples from Germany. The *in-silico* test against 20 commercially relevant non-target scallop species predicted that cross-species amplification of our SSPs is unlikely, even with its closest living relative, *A. ventricosus* [76,77]. This was confirmed by our *in-vitro* analysis, which showed no cross-amplification in DNA samples of *A. ventricosus*. Therefore, we expect that the SSPs for *A. purpuratus* will not cross-react with other more distantly related scallop species not included in our *in-vitro* assays. However, further analysis using DNA from additional scallop species will be necessary to validate the *in-silico* results obtained herein.

The high specificity of the Peruvian scallop’s SSPs was further demonstrated through a novel eDNA assay, which represents the first effort to explore the potential of single-species eDNA-based biomonitoring technology in Peruvian marine ecosystems. The scallop eDNA assay successfully detected the presence of *A. purpuratus* in all eDNA sampling sites where it is known to occur. During the collection of bottom eDNA samples from the Parachique station in Sechura Bay, we observed wild stocks of *A. purpuratus* and *A. ventricosus* coexisting in the same area. Nevertheless, our eDNA assay only detected the target scallop species, showing no signs of cross-reactivity with the eDNA of its congeneric relative *A. ventricosus*. This result emphasizes the high specificity of our assay, making it a valuable tool for discovering new beds of Peruvian scallops or for gaining further insights into population dynamics during extreme atmospheric conditions, such as the El Niño-Southern Oscillation (ENSO), which is known to impact the production and dispersal of Peruvian scallop larvae significantly [78].

Due to its benthic nature, we initially expected to find higher concentrations of *A. purpuratus* eDNA in bottom samples. However, our results from the Parachique station showed similar eDNA amounts in bottom, midwater, and surface samples (see Table 3). One possible reason for this is that all eDNA samples from the Parachique station were collected in shallow waters, specifically at depths ranging from 0 to 6 meters, which likely resulted in minimal vertical stratification. Interestingly, the slightly higher eDNA concentrations observed in midwater and surface samples might be attributed to the presence of planktonic gametes and larvae in the water column. This could be a result of spawning activity occurring days before and/or during the collection of water samples. The eDNA samples from the Parachique station (Sechura Bay) were collected in October 2017, which coincides with the peak spring spawning period (September to November) reported for the Peruvian scallop populations in Sechura Bay [79]. However, we cannot dismiss the possibility that restocking activities in nearby bottom culture sites contributed to the eDNA levels observed in our results, as natural horizontal dispersion of eDNA [80] shed by newly introduced spat may have influenced our findings. A strong Coastal El Niño event took place from January to April 2017 [70,81], causing high mortality rates among *A. purpuratus* populations in Sechura Bay. In an effort to recover from the substantial economic losses triggered by this mortality event, some scallop producers may have restocked their farms using wild or hatchery-produced spat. To better understand the spawning dynamics of Peruvian scallop populations, further studies involving repeated temporal sampling from wild scallop population sites (without aquaculture activity) and supported by quantitative assessment of gamete production and mesocosm experiments are necessary to confirm the effectiveness of our eDNA assay in identifying space-temporal spawning events.

The Patagonian longfin squid, *D. gahi*, is targeted by artisanal fisheries in Argentina, Chile, and Peru [82], and it is exported to several countries around the world [73,83]. In several nations, *D. hagi* (family Loliginidae) and *Dosidicus gigas* (family Ommastrephidae) are often sold under the generic terms “squid” or “*calamar*” in Spanish-speaking countries. However, *D. gigas* is of lower economic value and is available year-round, making it a suitable substitute for the more expensive Loliginidae squids [84,85]. The specificity of the SSP set DOGA136F/R targeting *D. hagi* was confirmed through *in-silico* and *in-vitro* specificity analyses performed on *D. hagi* samples. The *in-vitro* analysis included tests against four non-target squid species of commercial value: *Doryteuthis opalescens*, *Lolliguncula diomedeae*, *Dosidicus gigas*, and *Todarodes pacificus*. Considering all these results, the squid detection assay presented here will provide a rapid and cost-effective method for authenticating *D. hagi* products.

In Peru, several species from the family Paralichtyidae are sold simply as “*lenguado*” (Spanish for flounder) [38,41]. Among these, two significant Peruvian flatfish species of commercial interest are the fine flounder (*P. adspersus*) and the sole flounder (*E. ectenes*). The fine flounder is particularly noteworthy as it supports the largest Peruvian artisanal flatfish fishery and is the most sought-after and expensive flounder species [86], which has been reported to be a target of mislabeling [43,46]. The *in-vitro* PCR test results against 32 non-target species (including 6 commercially important flatfish species) demonstrated that our primer set PAAD165F/R is specific for *P. adspersus*. Similarly, the *in-vitro* results of the primer set ETROP162F/R showed that it reacted only with DNA from *E. ectenes*, though it is also anticipated to hybridize with DNA from its congener *E. crossotus*, as previously mentioned in the Results section. Furthermore, the *in-silico* validation against 10 congeneric *Paralichthys* species of global commercial value indicated that cross-species reactions are unlikely due to the presence of several mismatches at the 3’ end of both primers in non-target species (see panel C in S4 Fig and S6 File). This suggests that our assay may be useful in international markets. However, additional *in-vitro* analysis involving gDNA from other *Paralichthys* species will be necessary to confirm our *in-silico* findings.

Our results also demonstrated that the SSPs for *P. adspersus* can accurately identify different early life stages of this species. Further analyses using unsorted plankton samples are required to evaluate the effectiveness of the SSP set PAAD165F/R in detecting early life stages of the target species amidst a background of plankton community. Molecular assays for the accurate identification of early life stages of commercially important fish species can aid in locating spawning grounds and understanding seasonal variations in spawning activity [87]. Additionally, these assays can help during the collection of wild eggs and fry for farming purposes, reducing the risk of unintentionally rearing non-target species [88].

Designing efficient SSPs to differentiate closely related species can be challenging, particularly when the sequence differences in the primer hybridization region are limited to just one or two positions. Mismatches involving purine bases (i.e., A-A, A-G, G-G, or G-A) and pyrimidine bases (i.e., C-C) are known as critical mismatches, which have the most significant detrimental effects when they occur at the primer’s 3’ terminal [11,12]. To enhance specificity and reduce nonspecific amplifications, other variables can be adjusted. These include using lower primer concentrations, conducting fewer PCR cycles, and raising the annealing temperature as much as possible [89,90]. Additionally, using commercial PCR kits that contain ammonium sulfate in their buffer system is highly recommended, as this can improve primer specificity, decrease the likelihood of primer dimer formation, and reduce the yield of non-specific PCR products [56]. In our study, we demonstrated that punctual mutations and indels present at the 3’ end of the primer’s hybridization region in non-target congeneric species, such as *P. humeralis* and *P. callaensis*, combined with high annealing temperatures (61 °C and 62 °C, respectively) and low primer concentrations (0.1 µM and 0.15 µM, respectively) can effectively hinder cross-species reactions. The two *Paralabrax* species targeted in our study support important artisanal fisheries in Peru [91], where they are frequently marketed as “*cabrilla*” [38] (authors’ personal observation). We were able to standardize a duplex PCR-SSPs assay that combined the discriminatory power of two SSP sets, PACA163F/R (targeting *P. callaensis*) and PAHU288F/R (targeting *P. humeralis*), into a single reaction. This method allowed us to quickly and accurately identify cooked samples from both target species.

We successfully developed a second duplex PCR that effectively identifies two non-related fish species marketed under the common name “*ojo de uva*” (grape-eye): *H. macrophthalmos* (Malakichthyidae) and *S. haedrichi* (Centrolophidae). The latter species is also known as “*mocosa*”, “*cojinoba del norte*”, and “*cojinoba mocosa*” [41,69]. In some regions of northern Peru (e.g., La Libertad and Lambayeque regions), the commercial name “*ojo de uva*” is also attributed to *S. haedrichi* (authors’ personal observations). This does not necessarily imply mislabeling or species substitution but may reflect a case of synonymy with the common name for *H*. *macrophthalmos*. In this regard, our duplex assay, which targets both species sold as “*ojo de uva*”, can be effectively applied for their simultaneous differentiation. In Peru, *H. macrophthalmos* is considered a luxury commodity, with a tight supply and in high demand, particularly from high-end restaurants. Historical catch records indicate significant numbers for this species in 1987 and 1996; however, catches have drastically declined since 2014, suggesting overexploitation [44,92]. To support the recovery of *H*. *macrophthalmos* populations, it is essential to implement further management measures, such as temporary, spatial-temporal, or permanent closed seasons. In this context, our SSPs for *H*. *macrophthalmos* would serve as a powerful tool to detect illegal commercialization of this species.

Groupers (Epinephelidae) and grunts (Haemulidae) represent two fish groups of high economic value and in high demand, making them major targets for substitution [38,43,46]. Herein, we successfully developed SSP assays for one grouper, *Alphestes immaculatus*, and one grunt species, *Anisotremus interruptus*. Given that several species belonging to the family Epinephelidae (e.g., *Alphestes* spp., *Epinephelus* spp., *Mycteroperca* spp.) are commonly sold under the label “*mero*”, our assay for identifying *A. immaculatus* represents an important tool for authenticating processed samples labeled as “*mero*”. In Peru, *A. interruptus* is known as “*burrito*” or “*chita dorada*” [41,69], and more recently, it has been referred to as “*berrugata*” in commercial venues in the Piura region [44] (authors’ personal observation). Notably, “*berrugata*” is also the market name for the Pacific tripletail *Lobotes pacifica* [41,69]. Our *in-vitro* validation included fresh and cooked samples of *A. interruptus*, and the results showed 100% accuracy and high specificity, with no cross-reactions with the DNA from related or unrelated fish species, including *L. pacifica*. The identification assay for *A. interruptus* presented herein will enhance the rapid authentication of commercial samples labeled as “*berrugata*”.

## Conclusions, recommendations, and future perspectives

The seafood industry is experiencing rapid global growth, which increases the need for effective fishery management measures and strong regulatory systems supported by modern and reliable molecular identification tools. In response to the lack of species-specific identification assays for Peruvian seafood species, we designed and validated novel SSPs targeting 10 important marine species from the Eastern Pacific. We demonstrated the robustness, versatility, and specificity of our novel SSP assays through the rapid and accurate identification of target species in marine eDNA samples, as well as in fresh and processed commercial products. Our results include publicly available primer sequences and fully validated molecular protocols, which are ready for use by regulatory agencies, law enforcement bodies, research institutes, universities, and aquaculture corporations. This provides essential support for seafood certification programs, the future implementation of regulatory policies, traceability protocols, marine research, and aquaculture production.

Additionally, we introduced the first species-specific eDNA assay developed in Peru for a marine species, marking a significant advancement for the assessment of the highly economically important Peruvian scallop. Our eDNA assay successfully detected the presence of *A. purpuratus* at all sampling stations where this species is known to occur. The environmental DNA technique is an emergent technology that enhances traditional methods for estimating biomass or species abundance of various organisms. Moving forward, further efforts should be directed toward developing more eDNA assays for detecting and estimating the abundance of priority Peruvian marine resources, including protected, endangered, overexploited, commercially important, and invasive species. In this context, our novel SSPs targeting fish species can be beneficial for evaluating additional eDNA assays.

There is an urgent need to address several issues related to low taxonomic resolution during fish landings, including mislabeling, illegal fishing, weak regulations, a lack of traceability systems, and ineffective management of some Peruvian fishery resources. To tackle these challenges, initial efforts should focus on standardizing market names by creating an official list of unique commercial names. This recommendation has been made in the past [38,43,46] but has yet to be implemented. Additionally, we need to enforce fishery regulations, such as permanent or reproductive closed seasons, to ensure the recovery of overexploited resources like *H. macrophthalmos*. Finally, developing further molecular assays for seafood authentication and implementing eDNA surveillance programs will significantly aid efforts to combat overexploitation, illegal fishing, and mislabeling. These measures are essential for better management of our marine resources.

## Supporting information

S1 Table Non-target fish and shellfish species used in the interspecific validation of the species-specific primers. GenBank and BOLD accessions are written between parentheses and brackets, respectively. Sampling venues correspond to FLS: fish landing site, MK: market, SMK: supermarket, and WFM: wholesale fish market.
(DOCX)

S2 Table Primers used for DNA barcoding identification of the voucher specimens obtained in this study. (DOCX)

S3 Table List of all the DNA sequences generated in this study. (DOCX)

S1 Fig PCR amplification protocols for the 10 seafood species targeted in this study. (PDF)

S2 Fig Direct qPCR and standard PCR assays for the detection of commercial samples of *Argopecten purpuratus.* (TIF)

S3 Fig *In-silico* analysis of the primer set ARGOF/ARPU129R in *A. purpuratus* sequences. (PDF)

S4 Fig *In-silico* analysis against non-target species of the species-specific primers for A: *Argopecten purpuratus*, B: *Doryteuthis gahi*, C: *Paralichthys adspersus*, D: *Paralabrax callaensis*, and E: *P. humeralis.* (TIF)

S5 Fig *In-silico* analysis of the species-specific primers for A: *Etropus ectenes*, B: *Hemilutjanus macrophthalmos*, C: *Schedophilus haedrichi*, D: *Alphestes immaculatus*, and E: *Anisotremus interruptus.* (TIF)

S1 File Primer binding region of the SSP set for *Argopecten purpuratus* in all available DNA sequences of the target species in FASTA format. The FASTA file can be found in OSF: https://osf.io/h8ya9/files/osfstorage.
(FASTA)

S2 File Primer binding region of the SSP set for *Argopecten purpuratus* in 20 scallop species in FASTA format. The FASTA file can be found in OSF: https://osf.io/h8ya9/files/osfstorage.
(FASTA)

S3 File Primer binding region of the SSP set for *Doryteuthis gahi* in all available DNA sequences of the target species in FASTA format. The FASTA file can be found in OSF: https://osf.io/h8ya9/files/osfstorage.
(FASTA)

S4 File Primer binding region of the SSP set for *Doryteuthis gahi* in all available DNA sequences of non-target species *Dosidicus gigas* in FASTA format. The FASTA file can be found in OSF: https://osf.io/h8ya9/files/osfstorage.
(FASTA)

S5 File Primer binding region of the SSP set for *Paralichthys adspersus* in all available DNA sequences of the target species in FASTA format. The FASTA file can be found in OSF: https://osf.io/h8ya9/files/osfstorage.
(FASTA)

S6 File Primer binding region of the SSP set for *Paralichthys adspersus* in 10 congeneric species in FASTA format. The FASTA file can be found in OSF: https://osf.io/h8ya9/files/osfstorage.
(FASTA)

S7 File Primer binding region of the SSP set for *Etropus ectenes* in all available DNA sequences of the target species in FASTA format. The FASTA file can be found in OSF: https://osf.io/h8ya9/files/osfstorage.
(FASTA)

S8 File Primer binding region of the SSP set for *Paralabrax callaensis* in all available DNA sequences of the target species in FASTA format. The FASTA file can be found in OSF: https://osf.io/h8ya9/files/osfstorage.
(FASTA)

S9 File Primer binding region of the SSP set for *Paralabrax humeralis* in all available DNA sequences of the target species in FASTA format. The FASTA file can be found in OSF: https://osf.io/h8ya9/files/osfstorage.
(FASTA)

S10 File Primer binding region of the SSP set for *Hemilutjanus macrophthalmos* in all available DNA sequences of the target species in FASTA format. The FASTA file can be found in OSF: https://osf.io/h8ya9/files/osfstorage.
(FASTA)

S11 File Primer binding region of the SSP set for *Schedophilus haedrichi* in all available DNA sequences of the target species in FASTA format. The FASTA file can be found in OSF: https://osf.io/h8ya9/files/osfstorage.
(FASTA)

S12 File Primer binding region of the SSP set for *Alphestes immaculatus* in all available DNA sequences of the target species in FASTA format. The FASTA file can be found in OSF: https://osf.io/h8ya9/files/osfstorage.
(FASTA)

S13 File Primer binding region of the SSP set for *Anisotremus interruptus* in all available DNA sequences of the target species in FASTA format. The FASTA file can be found in OSF: https://osf.io/h8ya9/files/osfstorage.
(FASTA)
