# Supplementary material for: Development and validation of versatile species-specific primer assays for eDNA monitoring and authentication of 10 commercially important Peruvian marine species
Source: PLoS One. 2025 Jul 2;20(7):e0313181. doi: 10.1371/journal.pone.0313181 (PMC12221000; doi:10.1371/journal.pone.0313181)
Supplement: S1 Table — GenBank and BOLD accessions are written between parentheses and brackets, respectively. Sampling venues correspond to FLS: fish landing site, MK: market, SMK: supermarket, and WFM: wholesale fish market. (DOCX) [file pone.0313181.s001.docx]

**Development and validation of versatile species-specific primer assays for eDNA monitoring and authentication of 10 commercially important Peruvian marine species**

Alan Marín, Ruben Alfaro, Lorenzo E. Reyes-Flores, Claudia Ingar, Luis E. Santos-Rojas, Irina B. Alvarez-Jaque, Karen Rodríguez-Bernales, Cleila Carbajal, Angel Yon-Utrilla, Eliana Zelada-Mázmela

**S1 Table.** Fish and shellfish species used for non-cross species validation of the species-specific primers. GenBank and BOLD accessions are written between parenthesis and brackets respectively. Sampling venues correspond to FLS: fish landing site, MK: market, SMK: supermarket, and WFM: wholesale fish market

| **Code** | **Scientific name** | **Common name English/Spanish** | **Family** | **Orden** | **(GenBank)/**  **[BOLD] accession** | **Sampling site** | **Reference** |
| --- | --- | --- | --- | --- | --- | --- | --- |
| **FISH** | | | | | | | |
| **1** | *Trachinotus paitensis* | Paloma pompano / Pampanito | Carangidae | Carangiformes | (PP092957) | Buenos Aires MK (Ancash, Peru) | This study |
| **2** | *Coryphaena hippurus* | Dolphinfish / Perico | Coryphaenidae |  | (MH194449) | La Cruz FLS (Tumbes, Peru) | Marín et al. (2018) |
| **3** | *Xiphias gladius* | Swordfish / Pez espada | Xiphiidae |  | (MH194466) | Zorritos FLS (Tumbes, Peru) | Marín et al. (2018) |
| **4** | *Kajikia audax* | Striped marlin / Merlin | Istiophoridae |  | (MH194486) | Cancas FLS (Tumbes, Peru) | Marín et al. (2018) |
| **5** | *Calamus brachysomus* | Pacific porgy / Marotilla | Sparidae | Eupercaria *incertae sedis* | (PP092958) | José Olaya WFM (Piura, Peru) | This study |
| **6** | *Lobotes pacifica* | Pacific tripletail / Viuda | Lobotidae | Acanthuriformes | (MN839526) | Municipal MK Máncora (Piura, Peru) | Marín et al. (2021) |
| **7** | *Paralonchurus peruanus* | Peruvian banded croaker / Suco | Sciaenidae | Eupercaria *incertae sedis* | (PP092959) | Buenos Aires MK (Ancash, Peru) | This study |
| **8** | *Cheilotrema fasciatum* | Arnillo drum / Burro |  |  | (MN839519) | Las Sirenas MK (Ancash, Peru) | Marín et al. (2021) |
| **9** | *Anisotremus scapularis* | Peruvian grunt/ Chita | Haemulidae |  | (MN839516) | Las Sirenas MK (Ancash, Peru) | Marín et al. (2021) |
| **10** | *Anisotremus interruptus* | Burrito grunt / Berrugata |  |  | (MN839517) | José Olaya WFM (Piura, Peru) | Marín et al. (2021) |
| **11** | *Paralabrax callaensis* | Southern rock bass / Perela | Serranidae | Perciformes | (MN839525) | Modelo MK (Tumbes, Peru) | Marín et al. (2021) |
| **12** | *Paralabrax humeralis* | Peruvian rock seabass / Cagalo |  |  | (MN839526) | Buenos Aires MK (Ancash, Peru) | Marín et al. (2021) |
| **13** | *Hemilutjanus macrophthalmos* | Grape-eye seabass / Ojo de uva | Malakichthydae | Acropomatiformes | (MN839520) | José Olaya WFM (Piura, Peru) | Marín et al. (2021) |
| **14** | *Mycteroperca xenarcha* | Broomtail grouper / Mero murique | Epinephelidae | Perciformes | (MN839523) | José Olaya WFM (Piura, Peru) | Marín et al. (2021) |
| **15** | *Hyporthodus acanthistius* | Rooster hind / Mero colorado |  |  | (MN839521) | Modelo MK (Tumbes, Peru) | Marín et al. (2021) |
| **16** | *Alphestes immaculatus* | Pacific mutton hamlet / Mero rojo |  |  | (PP092955) | Santa Rosa WFM (Lambayeque, Peru) | This study |
| **17** | *Acanthistius pictus* | Brick seabass / Cherlo | Anthiadidae |  | (MN839524) | Santa Rosa WFM (Lambayeque, Peru) | Marín et al. (2021) |
| **18** | *Hyporthodus niphobles* | Star-studded grouper / Mero manchado | Epinephelidae |  | (PP092960) | Modelo MK (Tumbes, Peru) | This study |
| **19** | *Epinephelus analogus* | Spotter grouper / Mero moteado |  |  | (PP092961) | José Olaya WFM (Piura, Peru) | This study |
| **20** | *Schedophilus haedrichi* | Mocosa ruff / Mocosa | Centrolophidae | Scombriformes | (MN839529) | Mayorista MK (La Libertad, Trujillo) | Marín et al. (2021) |
| **21** | *Prionotus stephanophrys* | Lumptail searobin / Falso volador | Triglidae | Perciformes | (MN839515) | Modelo MK (Tumbes, Peru) | Marín et al. (2021) |
| **22** | *Opisthonema medirastre* | Middling thread herring / Machete | Dorosomatidae | Clupeiformes | (MN839514) | La Cruz FLS (Tumbes, Peru) | Marín et al. (2021) |
| **23** | *Odontesthes regia* | Chilean silverside / Pejerrey | Atherinopsidae | Atheriniformes | (MN839513) | Buenos Aires MK (Ancash, Peru) | Marín et al. (2021) |
| **24** | *Merluccius peruanus* | Peruvian hake / Merluza | Merlucciidae | Gadiformes | (PP092962) | Buenos Aires MK (Ancash, Peru) | This study |
| **25** | *Mugil cephalus* | Lisa | Mugilidae | Mugiliformes | (PP092963) | Buenos Aires MK (Ancash, Peru) | This study |
| **26** | *Hippoglossina tetrophthalma* | Fourspot flounder / Lenguado de cuatro ocelos | Paralichthyidae | Pleuronectiformes | MN839530) | Santa Rosa WFM (Lambayeque, Peru) | Marín et al. (2021) |
| **27** | *Sphyrna zygaena* | Smooth hammerhead / Tiburón martillo | Sphyrnidae | Carcharhiniformes | (MH194504) | Cancas FLS (Tumbes, Peru) | Marín et al. (2018) |
| **28** | *Ancylopsetta dendritica* | Three-spot flounder / Lenguado tres ocelos | Paralichthyidae | Pleuronectiformes | (PP092964) | José Olaya WFM (Piura, Peru) | This study |
| **29** | *Cyclopsetta querna* | Toothed flounder / Lenguado con caninos |  |  | (PP092965) | La Cruz FLS (Tumbes, Peru) | This study |
| **30** | *Paralichthys woolmani* | Speckled flounder / Lenguado |  |  | (MN880555, MN880580, MN880581, MN880582) | La Tortuga FLS (Piura, Peru);  Puerto Pizarro FLS (Tumbes, Peru) | Marín et al. (2022) |
| **31** | *Symphurus chabanaudi* | Chabanaud's tonguefish  / Lengüeta punta fina | Cynoglossidae |  | (PP092966) | Grau FLS (Tumbes, Peru) | This study |
| **BIVALVES** | | | | | | | |
| **1** | *Argopecten ventricosus* | Pacific calico scallop / Concha panameña | Pectinidae | Pectinida | (PP087194 to PP087213) | Sechura Bay n=20 (Piura, Peru) | This study |
|  |  |  |  |  | (PP087214 to PP087220) | Santa Elena n=7 (Ecuador) |  |
| **2** | *Pteria sterna* | Pacific wing-oyster / Concha perlífera | Pteriidae | Ostreida | [PEMAR_I1504] | La Cruz FLS (Tumbes, Peru) | *Rodríguez-Bernales, KD (2021) |
| **3** | *Striostrea prismatica* | Stone oyster / Ostra nativa | Ostreidae |  | [PEMAR_I1464] | La Cruz FLS (Tumbes, Peru) | *Rodríguez-Bernales, KD (2021) |
| **4** | *Atrina maura* | Maura pen shell / Concha lampa | Pinnidae |  | [PEMAR_I1469] | La Cruz FLS (Tumbes, Peru) | *Rodríguez-Bernales, KD (2021) |
| **5** | *Gari solida* | Pacific clam / Almeja | Psammobiidae | Cardiida | [PEMAR_I0207] | Tortugas Bay (Ancash, Peru) | *Rodríguez-Bernales, KD (2021) |
| **6** | *Perumytilus purpuratus* | Purple mussel / Chorito | Mytilidae | Mytilida | [PEMAR_I0305] | Melchorita Beach (Lima, Peru) | *Rodríguez-Bernales, KD (2021) |
| **7** | *Aulacomya atra* | Chilean ribbed mussel / Choro |  | Mytilida | [PEMAR_I1985] | Laguna Grande MK (Pisco, Peru) | *Rodríguez-Bernales, KD (2021) |
| **CEPHALOPODS** | | | | | | | |
| **1** | *Doryteuthis opalescens* | Opalescent Inshore squid / Calamar de California | Loliginidae | Myopsida | (PQ459853, PQ459854) | SMK n=2 (La Libertad, Peru)  Product imported from USA | This study |
| **2** | *Lolliguncula diomedeae* | Dart squid / Calamar dardo |  |  | (PQ459849, PQ459850) | Puerto Pizarro FLS n=2 (Tumbes, Peru) | This study |
| **3** | *Dosidicus gigas* | Jumbo flying squid / Pota | Ommastrephidae | Oegopsida | [PEMAR_I1675] | Gildemeister pier fish landing (Ancash, Peru)  Modelo MK n=1 (Tumbes, Peru)  Buenos Aires WFM n=1 (La Libertad, Peru) | This study |
| **4** | *Todarodes pacificus* | Japanese flying squid / Calamar volador japonés |  |  | (PQ459855) | SMK n=2 (Nagoya, Japan) | This study |

*Rodríguez Bernales, K. D. (2021). Identificación molecular de especies de bivalvos, cefalópodos y poliplacóforos mediante el código de barras de ADN, para la conservación de la biodiversidad del litoral peruano. Tesis Universidad Nacional del Santa
