# Supplementary material for: Development and validation of versatile species-specific primer assays for eDNA monitoring and authentication of 10 commercially important Peruvian marine species
Source: PLoS One. 2025 Jul 2;20(7):e0313181. doi: 10.1371/journal.pone.0313181 (PMC12221000; doi:10.1371/journal.pone.0313181)
Supplement: S2 Table — (DOCX) [file pone.0313181.s002.docx]

**Development and validation of versatile species-specific primer assays for eDNA monitoring and authentication of 10 commercially important Peruvian marine species**

Alan Marín, Ruben Alfaro, Lorenzo E. Reyes-Flores, Claudia Ingar, Luis E. Santos-Rojas, Irina B. Alvarez-Jaque, Karen Rodríguez-Bernales, Cleila Carbajal, Angel Yon-Utrilla, Eliana Zelada-Mázmela

**S2 Table.** Primers used for barcoding identification of voucher specimens obtained in this study

| **Gene target** | **Primer name** | **Direction** | **Sequence (5'-3')** | **Ta (˚C)** | **Target species group** | **Source** |
| --- | --- | --- | --- | --- | --- | --- |
| 16S | PurvenF | Forward | CTAAGGGGTGAAGGTAATAATG | 58-60 | Scallops | This study |
|  | 16Sbr-H | Reverse | CCGGTCTGAACTCAGATCACGT |  |  | Palumbi et al. (1991) |
|  | 16Sar-L | Forward | CGCCTGTTTATCAAAAACAT |  |  |  |
| COI | FishF1 | Forward | TCAACCAACCACAAAGACATTGGCAC | 50-54 | Fish | Ward et al. (2005) |
|  | FishR1 | Reverse | TAGACTTCTGGGTGGCCAAAGAATCA |  |  |  |
|  | LCO1490 | Forward | \| GGTCAACAAATCATAAAGATATTGG \| \| --- \| \|  \| | 52 | Universal | Folmer et al. (1994) |
|  | HCO2198 | Reverse | \| TAAACTTCAGGGTGACCAAAAAATCA \| \| --- \| |  |  |  |
| 12S | MiFish-U-F | Forward | GTCGGTAAAACTCGTGCCAGC | 60 | Fish | Miya et al. (2015) |
|  | MiFish-U-R | Reverse | CATAGTGGGGTATCTAATCCCAGTTTG |  |  |  |
