# Supplementary material for: Development and validation of versatile species-specific primer assays for eDNA monitoring and authentication of 10 commercially important Peruvian marine species
Source: PLoS One. 2025 Jul 2;20(7):e0313181. doi: 10.1371/journal.pone.0313181 (PMC12221000; doi:10.1371/journal.pone.0313181)
Supplement: S3 Table — (DOCX) [file pone.0313181.s003.docx]

**Development and validation of versatile species-specific primer assays for eDNA monitoring and authentication of 10 commercially important Peruvian marine species**

Alan Marín, Ruben Alfaro, Lorenzo E. Reyes-Flores, Claudia Ingar, Luis E. Santos-Rojas, Irina B. Alvarez-Jaque, Karen Rodríguez-Bernales, Cleila Carbajal, Angel Yon-Utrilla, Eliana Zelada-Mázmela

**S3 Table.** DNA sequences generated in this study.

| Species | n | Source | Gen | Primers | GenBank |
| --- | --- | --- | --- | --- | --- |
| *Argopecten purpuratus* | 34 | Adductor muscle | 16S | Purven+16Sbr-H | PP087160 to PP087193 |
| *Argopecten ventricosus* | 27 | Adductor muscle | 16S | Purven+6Sbr-H | PP087193 to PP087220 |
| *Paralabrax callaensis* | 10 | Spine | 16S | 16Sbr-L+16Sbr-H | PQ459826 to PQ459835 |
| *Paralabrax humeralis* | 10 | Spine | 16S | 16Sbr-L+16Sbr-H | PQ459836 to PQ459845 |
| *Alphestes immaculatus* | 3 | Spine | 16S | 16Sbr-L+16Sbr-H | PQ459846 to PQ459848 |
| *Lolliguncula diomedeae* | 2 | Tentacle | 16S | 16Sbr-L+16Sbr-H | PQ459849 to PQ459850 |
| *Doryteuthis gahi* | 2 | Tentacle | 16S | 16Sbr-L+16Sbr-H | PQ459851 to PQ459852 |
| *Doryteuthis opalescens* | 2 | Tentacle | 16S | 16Sbr-L+16Sbr-H | PQ459853 to PQ459854 |
| *Todarodes pacificus* | 1 | Tentacle | 16S | 16SARL | PQ459855 |
| *Haemulon scudderii* | 2 | Spine | COI | FishF1+ FishR1 | PQ459803 to PQ459804 |
| *Schedophilus haedrichi* | 5 | Spine | COI | FishF1+ FishR1 | PQ459805 to PQ459809 |
| *Hemilutjanus macrophthalmos* | 3 | Spine | COI | FishF1+ FishR1 | PQ459810 to PQ459812 |
| *Prionotus stephanophrys* | 2 | Spine | COI | FishF1+ FishR1 | PQ459813 to PQ459814 |
| *Paralichthys adspersus* | 11 | Spine | COI | FishF1+ FishR1 | PQ459815 to PQ459825 |
| *Paralichthys adspersus* | 1 | Spine | COI | FishF1+ FishR1 | PP092941 |
| *Etropus ectenes* | 1 | Spine | COI | FishF1+ FishR1 | PP092942 |
| *Paralabrax callaensis* | 5 | Spine | COI | FishF1+ FishR1 | PP092943 to PP092947 |
| *Paralabrax humeralis* | 5 | Spine | COI | FishF1+ FishR1 | PP092948 to PP092952 |
| *Hemilutjanus macrophthalmos* | 1 | Spine | COI | FishF1+ FishR1 | PP092953 |
| *Schedophilus haedrichi* | 1 | Spine | COI | FishF1+ FishR1 | PP092954 |
| *Alphestes immaculatus* | 1 | Spine | COI | FishF1+ FishR1 | PP092955 |
| *Anisotremus interruptus* | 1 | Spine | COI | FishF1+ FishR1 | PP092956 |
| *Trachinotus paitensis* | 1 | Spine | COI | FishF1+ FishR1 | PP092957 |
| *Calamus brachysomus* | 1 | Spine | COI | FishF1+ FishR1 | PP092958 |
| *Paralonchurus peruanus* | 1 | Spine | COI | FishF1+ FishR1 | PP092959 |
| *Hyporthodus niphobles* | 1 | Spine | COI | FishF1+ FishR1 | PP092960 |
| *Epinephelus analogus* | 1 | Spine | COI | FishF1+ FishR1 | PP092961 |
| *Merluccius gayi* | 1 | Spine | COI | FishF1+ FishR1 | PP092962 |
| *Mugil cephalus* | 1 | Spine | COI | FishF1+ FishR1 | PP092963 |
| *Ancylopsetta dendritica* | 1 | Spine | COI | FishF1+ FishR1 | PP092964 |
| *Cyclopsetta querna* | 1 | Spine | COI | FishF1+ FishR1 | PP092965 |
| *Symphurus chabanaudi* | 1 | Spine | COI | FishF1+ FishR1 | PP092966 |
| *Doryteuthis gahi* | 1 | Tentacle | COI | LCO1490+ HCO2198 | PV562158 |
| eDNA | | | | | |
| *Argopecten purpuratus* | 11 | eDNA | 16S | ARGOF+ARPU129R | PP087144 to PP087154 |
| COOKED | | | | | |
| *Paralabrax callaensis* | 2 | Cooked muscle | 16S | PACA163F+ PACA163R | PP087227 and PPP087228 |
| *Paralabrax humeralis* | 2 | Cooked muscle | 16S | PAHU288F+ PAHU288R | PPP087229 and PV491563 |
| *Argopecten purpuratus* | 1 | Cooked muscle | 16S | ARGOF+ARPU129R | PV491562 |
| *Cyclopsetta querna* | 1 | Fried muscle | 12S | MiFish-U-F+ MiFish-U-R | PV505011 |
| *Paralichthys woolmani* | 1 | Fried muscle | 12S | MiFish-U-F+ MiFish-U-R | PV505012 |
| *Anisotremus interruptus* | 1 | Fried muscle | COI | ANIN246F+ ANIN246R | PV562156 |
| *Schedophilus haedrichi* | 1 | Marinated muscle | COI | SCHA244F+SCHA244R | PV562157 |
| *Dosidicus gigas* | 2 | Cooked tentacle | COI | LCO1490+ HCO2198 | PV562159 and PV562160 |
| *Cyclopsetta querna* | 1 | Fried muscle | COI | LCO1490+ HCO2198 | PV562161 |
| *Doryteuthis gahi* | 3 | Cooked tentacle | COI | DOGA136F+ DOGA136R | 5*, 10*, 11* |
| *Hemilutjanus macrophthalmos* | 1 | Stewed muscle | COI | HEMA122F+HEMA122R | 9* |

*These sequences are shown below because the GenBank database no longer accepts DNA sequences shorter than 150 nucleotides.

>*Doryteuthis gahi* 5

CGATGAGAAGGTTTATTAATAGAACGACTTTCTCTATTTGTTTGATCTGTTTTTATTACTGCTATTCTTCTCCTTCTTTCTCTCCCAGTACTAGCTGGTGCCATTACAATATTACTAACCGATCGAAACTTT

> *Doryteuthis gahi* 10

CGATGAGAAGGTTTATTAATAGAACGACTTTCTCTATTTGTTTGATCTGTTTTTATTACTGCTATTCTTCTCCTTCTTTCTCTCCCAGTACTAGCTGGTGCCATTACAATATTACTAACCGATCGAAACTTT

> *Doryteuthis gahi* 11

CGATGAGAAGGTTTATTAATAGAACGACTTTCTCTATTTGTTTGATCTGTTTTTATTACTGCTATTCTTCTCCTTCTTTCTCTCCCAGTACTAGCTGGTGCCATTACAATATTACTAACCGATCGAAACTTT

>*Hemilutjanus macrophthalmos* 9

TAACTTCATCACAACTATTATTAACATGAAACCGCCTGCATCTCCCAATATCAGACACCCTTATTTGTGTGAGCTGTTCTAATCACTGCCGTTCTTCTTCTAC
