# Supplementary material for: Development and validation of versatile species-specific primer assays for eDNA monitoring and authentication of 10 commercially important Peruvian marine species
Source: PLoS One. 2025 Jul 2;20(7):e0313181. doi: 10.1371/journal.pone.0313181 (PMC12221000; doi:10.1371/journal.pone.0313181)
Supplement: S1 Fig — (PDF) [file pone.0313181.s004.pdf]

Development and validation of versatile species-specific primer assays for eDNA monitoring and authentication of 10 commercially important Peruvian marine species

Alan Marín, Ruben Alfaro, Lorenzo E. Reyes-Flores, Claudia Ingar, Luis E. Santos-Rojas, Irina B. Alvarez-Jaque, Karen Rodríguez-Bernales, Cleila Carbajal, Angel Yon-Utrilla, Eliana Zelada-Mázmela

S1 Fig. Species-species primer sequences, master mix composition, and simplex and duplex PCR amplification protocols for the species identification of: A) *Argopecten purpuratus*, B) *Doryteuthis gahi*, C) *Paralichthys adspersus*, D) *Etropus ectenes*, E) *Paralabrax callaensis*, F) *Paralabrax humeralis*, G) *Hemilutjanus macrophthalmos*, H) *Schedophilus haedrichi*, I) *Alphestes immaculatus*, and J) *Anisotremus interruptus*.

The inset maps were reprinted from MapChart Online maps under a CC BY license, with permission from MapChart, original copyright 2025. The copyrights belong to MapChart, but according to the terms of use, the copyright holder does not need to apply for permission to use because it is free for academic publications, and can be used freely and commercially under the CC BY 4.0 license.

A *Argopecten purpuratus*

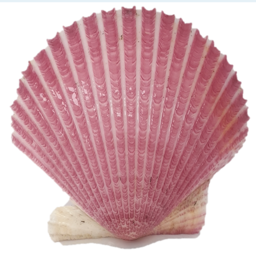

Argopecten purpuratus (Lamarck, 1819)

Common name: Peruvian scallop (*concha de abanico*)
Family: Pectinidae
Price at landing: US$1.5-3/kg

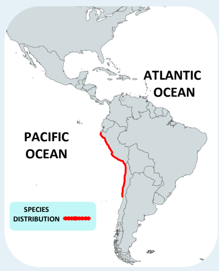

SPECIES-SPECIFIC PRIMERS					
NAME	SEQUENCE 5'-3'	SENSE	[FINAL] µM	SIZE bp	GENE
ARGOF	CTTCTGTCTCTAGCTTGTTTTAGTG	Forward	0.1-0.2	129	16S
ARPU129R	GCTAAGGGAAGTAACCTTCTAC	Reverse			

STANDARD PCR MIX	
PCR H_2O	3.6 µL
Premix 2X	5 µL
ARGOF (10 µM)	0.2 µL
ARPU129R (10 µM)	0.2 µL
DNA (10-20 ng/µL)	1 µL
TOTAL	10 µL

qPCR MIX	
PCR H_2O	7.6 µL
SYBR Green I Master 2X	10 µL
ARGOF (10 µM)	0.2 µL
ARPU129R (10 µM)	0.2 µL
DNA (10 ng/µL)	2 µL
TOTAL	20 µL

STANDARD PCR CYCLING PROTOCOL			
STEP	TEMP. °C	TIME	CYCLES
Denaturation	95	5 min	1
Denaturation	95	20 s	35
Hybridization	61	20 s	
Extension	72	20 s	
Final extension	72	7 min	1

qPCR CYCLING PROTOCOL			
STEP	TEMP. °C	TIME	CYCLES
Denaturation	95	5 min	1
Denaturation	95	15 s	40
Hybridization/ extension	61	1 min	

B *Doryteuthis gahi*

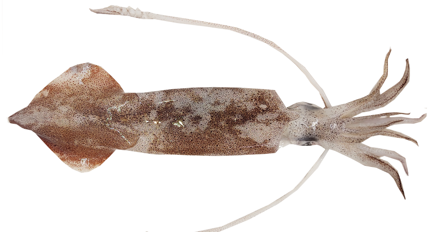

Doryteuthis gahi (d'Orbigny, 1835)

Common name: Patagonian longfin squid (*calamar común*)
Family: Loliginidae
Price at landing: US$1-5/kg

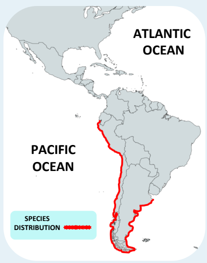

SPECIES-SPECIFIC PRIMERS					
NAME	SEQUENCE 5'-3'	SENSE	[FINAL] µM	SIZE bp	GENE
DOGA136F	GGCGATGAGAAGGTTTATT	Forward	0.2	136	COI
DOGA136R	CCAAAGTTTCGATCGGTTAGTAA	Reverse			

STANDARD PCR MIX	
PCR H_2O	4.1 µL
Premix 2X	5 µL
DOGA136F (10 µM)	0.2 µL
DOGA136R (10 µM)	0.2 µL
DNA (10-20 ng/µL)	0.5 µL
TOTAL	10 µL

CYCLING PROTOCOL			
STEP	TEMPERATURE °C	TIME	CYCLES
Denaturation	94	5 min	1
Denaturation	95	20 s	35
Hybridization	60	20 s	
Extension	72	20 s	
Final extension	72	7 min	1

C *Paralichthys adspersus*

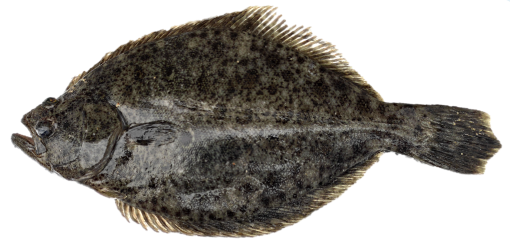

Paralicthys adspersus (Steindachner, 1867)

Common name: Fine flounder (*lenguado fino*)
Family: Paralichthyidae
Price at landing: US$3-10/kg

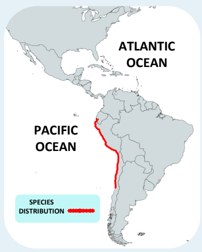

SPECIES-SPECIFIC PRIMERS					
NAME	SEQUENCE 5'-3'	SENSE	[FINAL] µM	SIZE bp	GENE
PAAD165F	ATACCAAGTCCCCCTATTTATC	Forward	0.1	165	COI
PAAD165R	TGTTGGTAGAGGATGGGATCA	Reverse			

STANDARD PCR MIX	
PCR H_2O	4.3 µL
Premix 2X	5 µL
PAAD165F (10 µM)	0.1 µL
PAAD165R (10 µM)	0.1 µL
DNA (10-20 ng/µL)	0.5 µL
TOTAL	10 µL

DUPLEX PCR MIX	
PCR H_2O	4.14 µL
Premix 2X	5 µL
PAAD165F (10 µM)	0.1 µL
PAAD165R (10 µM)	0.1 µL
MiFish-U-F (10 µM)	0.08 µL
MiFish-U-R (10 µM)	0.08 µL
DNA (10-20 ng/µL)	0.5 µL
TOTAL	10 µL

CYCLING PROTOCOL			
STEP	TEMPERATURE °C	TIME	CYCLES
Denaturation	94	5 min	1
Denaturation	95	20 s	35
Hybridization	60	20 s	
Extension	72	20 s	
Final extension	72	7 min	1

D *Etropus ectenes*

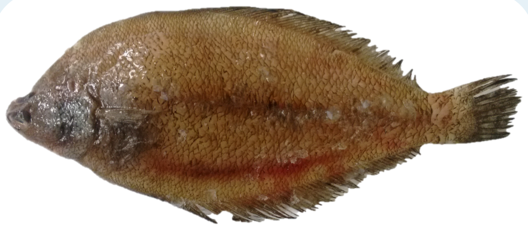

Etropus ectenes Jordan, 1889

Common name: Sole flounder (*lenguado boca chica*)
Family: Paralichthyidae
Price at landing: US$0.5-1/kg

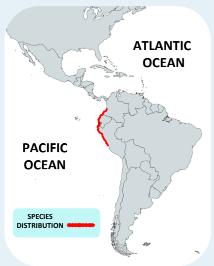

SPECIES-SPECIFIC PRIMERS					
NAME	SEQUENCE 5'-3'	SENSE	[FINAL] µM	SIZE bp	GENE
ETROP162F	TATCAACATGAAGCCCACAT	Forward	0.1	162	COI
ETROP162R	GGGTCAAAGAAGGTTGTGTTCAAA	Reverse			

STANDARD PCR MIX	
PCR H_2O	4.3 µL
Premix 2X	5 µL
ETROP162F (10 µM)	0.1 µL
ETROP162R (10 µM)	0.1 µL
DNA (10-20 ng/µL)	0.5 µL
TOTAL	10 µL

DUPLEX PCR MIX	
PCR H_2O	4.14 µL
Premix 2X	5 µL
ETROP162F (10 µM)	0.1 µL
ETROP162R (10 µM)	0.1 µL
MiFish-U-F (10 µM)	0.08 µL
MiFish-U-R (10 µM)	0.08 µL
DNA (10-20 ng/µL)	0.5 µL
TOTAL	10 µL

CYCLING PROTOCOL			
STEP	TEMPERATURE °C	TIME	CYCLES
Denaturation	94	5 min	1
Denaturation	95	20 s	35
Hybridization	60	20 s	
Extension	72	20 s	
Final extension	72	7 min	1

E *Paralabrax callaensis*

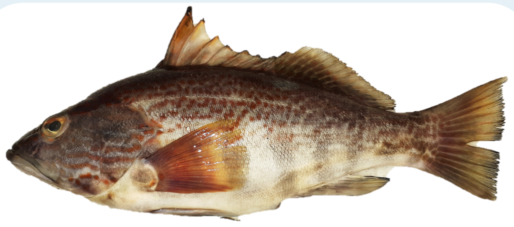

Paralabrax callaensis Starks, 1906

Common name: Southern rock bass (*cabrilla fina*)
Family: Serranidae
Price at landing: US$1.5-7/kg

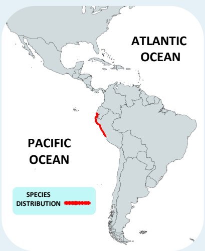

SPECIES-SPECIFIC PRIMERS					
NAME	SEQUENCE 5'-3'	SENSE	[FINAL] µM	SIZE bp	GENE
PACA163F	ACCCTATGAAGCTTTAGACACCAGA	Forward	0.1	163	16S
PACA163R	GCTCTGGGTTGTAAGAGAGTAAAA	Reverse			

STANDARD PCR MIX	
PCR H_2O	4.3 µL
Premix 2X	5 µL
PACA163F (10 µM)	0.1 µL
PACA163R (10 µM)	0.1 µL
DNA (10-20 ng/µL)	0.5 µL
TOTAL	10 µL

DUPLEX PCR MIX	
PCR H_2O	4.1 µL
Premix 2X	5 µL
PACA163F (10 µM)	0.1 µL
PACA163R (10 µM)	0.1 µL
MiFish-U-F (10 µM)	0.1 µL
MiFish-U-R (10 µM)	0.1 µL
DNA (10-20 ng/µL)	0.5 µL
TOTAL	10 µL

CYCLING PROTOCOL			
STEP	TEMPERATURE °C	TIME	CYCLES
Denaturation	94	5 min	1
Denaturation	95	20 s	33
Hybridization	61	20 s	
Extension	72	20 s	
Final extension	72	5 min	1

F *Paralabrax humeralis*

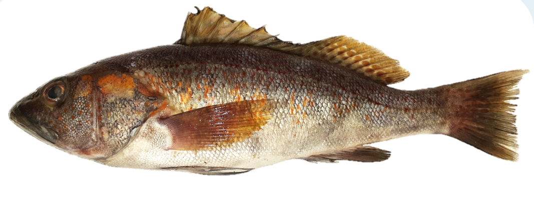

Paralabrax humeralis (Valenciennes, 1828)

Common name: Peruvian rock seabass (*cabrilla común*)
Family: Serranidae
Price at landing: US$3-8/kg

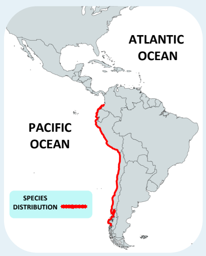

SPECIES-SPECIFIC PRIMERS					
NAME	SEQUENCE 5'-3'	SENSE	[FINAL] µM	SIZE bp	GENE
PAHU288F	CGCAATCACTTGTCCC	Forward	0.15	288	16S
PAHU288R	CTCTGGGTTGTAAGAGAGTTAAT	Reverse			

STANDARD PCR MIX	
PCR H_2O	4.2 µL
Premix 2X	5 µL
PAHU288F (10 µM)	0.15 µL
PAHU288R (10 µM)	0.15 µL
DNA (10-20 ng/µL)	0.5 µL
TOTAL	10 µL

DUPLEX PCR MIX	
PCR H_2O	4 µL
Premix 2X	5 µL
PAHU288F (10 µM)	0.15 µL
PAHU288R (10 µM)	0.15 µL
MiFish-U-F (10 µM)	0.1 µL
MiFish-U-R (10 µM)	0.1 µL
DNA (10-20 ng/µL)	0.5 µL
TOTAL	10 µL

CYCLING PROTOCOL			
STEP	TEMPERATURE °C	TIME	CYCLES
Denaturation	94	5 min	1
Denaturation	95	20 s	35
Hybridization	62	20 s	
Extension	72	20 s	
Final extension	72	7 min	1

G *Hemilutjanus macrophthalmos*

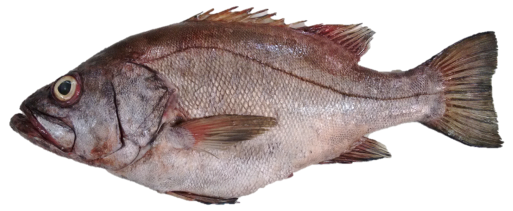

Hemilutjanus macrophthalmos (Tschudi, 1846)

Common name: Grape-eye seabass (*ojo de uva*)
Family: Malakichthyidae
Price at landing: US$5-7.7/kg

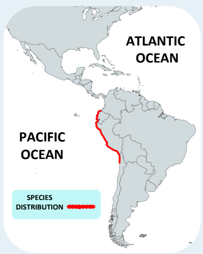

SPECIES-SPECIFIC PRIMERS					
NAME	SEQUENCE 5'-3'	SENSE	[FINAL] µM	SIZE bp	GENE
HEMA122F	AATCCTCGGGGCCATTAACTTCATC	Forward	0.2	122	COI
HEMA122R	AAAGTAGAAGAAGAACGGCAGTG	Reverse			

STANDARD PCR MIX	
PCR H_2O	3.6 µL
Premix 2X	5 µL
HEMA122F (10 µM)	0.2 µL
HEMA122R (10 µM)	0.2 µL
DNA (10-20 ng/µL)	1 µL
TOTAL	10 µL

DUPLEX PCR MIX	
PCR H_2O	3.9 µL
Premix 2X	5 µL
HEMA122F (10 µM)	0.2 µL
HEMA122R (10 µM)	0.2 µL
MiFish-U-F (10 µM)	0.1 µL
MiFish-U-R (10 µM)	0.1 µL
DNA (10-20 ng/µL)	0.5 µL
TOTAL	10 µL

CYCLING PROTOCOL			
STEP	TEMPERATURE °C	TIME	CYCLES
Denaturation	94	5 min	1
Denaturation	95	20 s	33
Hybridization	60	20 s	
Extension	72	20 s	
Final extension	72	5 min	1

H *Schedophilus haedrichi*

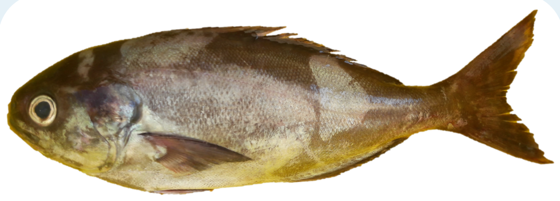

Schedophilus haedrichi Chirichigno F., 1973

Common name: Mocosa ruff (*cojinova mocosa*)
Family: Centrolophidae
Price at landing: US$4-5.5/kg

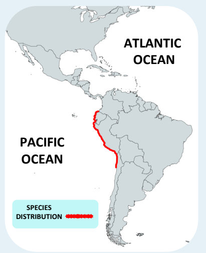

SPECIES-SPECIFIC PRIMERS					
NAME	SEQUENCE 5'-3'	SENSE	[FINAL] µM	SIZE bp	GENE
SCHA244F	AACTGGTTGAACAGTATATCCTCCT	Forward	0.1-0.2	244	COI
SCHA244R	AGCAGCTAGGACAGGGAGAGATAAT	Reverse			

STANDARD PCR MIX	
PCR H_2O	3.6 µL
Premix 2X	5 µL
SCHA244F (10 µM)	0.2 µL
SCHA244R (10 µM)	0.2 µL
DNA (10-20 ng/µL)	1 µL
TOTAL	10 µL

DUPLEX PCR MIX	
PCR H_2O	4.1 µL
Premix 2X	5 µL
SCHA244F (10 µM)	0.1 µL
SCHA244R (10 µM)	0.1 µL
MiFish-U-F (10 µM)	0.1 µL
MiFish-U-R (10 µM)	0.1 µL
DNA (10-20 ng/µL)	0.5 µL
TOTAL	10 µL

CYCLING PROTOCOL			
STEP	TEMPERATURE °C	TIME	CYCLES
Denaturation	94	5 min	1
Denaturation	95	20 s	35
Hybridization	60	20 s	
Extension	72	20 s	
Final extension	72	5 min	1

I *Alphestes immaculatus*

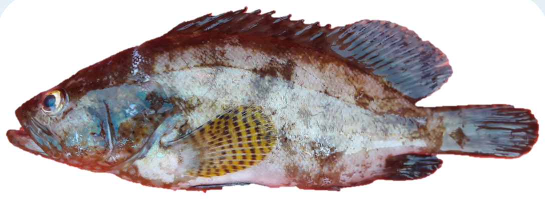

Alphestes immaculatus Breder, 1936

Common name: Pacific mutton hamlet (*merito rojo*)
Family: Epinephelidae
Price at landing: US$1.5-5.5/kg

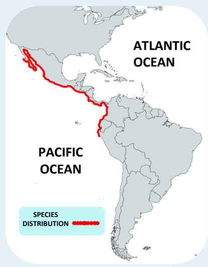

SPECIES-SPECIFIC PRIMERS					
NAME	SEQUENCE 5'-3'	SENSE	[FINAL] µM	SIZE bp	GENE
ALIM135F	GCGACACTAAAGCAGATCATAT	Forward	0.2	135	16S
ALIM135R	GTAGTACATTCGGTCCTTAT	Reverse			

STANDARD PCR MIX	
PCR H_2O	4.1 µL
Premix 2X	5 µL
ALIM135F (10 µM)	0.2 µL
ALIM135R (10 µM)	0.2 µL
DNA (10-20 ng/µL)	0.5 µL
TOTAL	10 µL

DUPLEX PCR MIX	
PCR H_2O	3.9 µL
Premix 2X	5 µL
ALIM135F (10 µM)	0.2 µL
ALIM135R (10 µM)	0.2 µL
MiFish-U-F (10 µM)	0.1 µL
MiFish-U-R (10 µM)	0.1 µL
DNA (10-20 ng/µL)	0.5 µL
TOTAL	10 µL

CYCLING PROTOCOL			
STEP	TEMPERATURE °C	TIME	CYCLES
Denaturation	94	5 min	1
Denaturation	95	20 s	35
Hybridization	60	20 s	
Extension	72	20 s	
Final extension	72	5 min	1

J *Anisotremus interruptus*

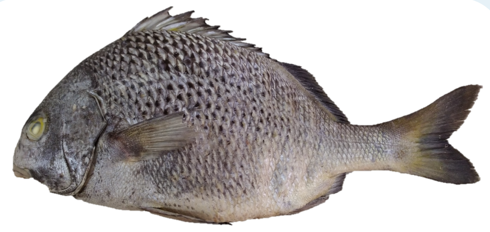

Anisotremus interruptus (Gill, 1862)

Common name: Burrito grunt (*Chita dorada*)
Family: Haemulidae
Price at landing: US$1.4-5.5/kg

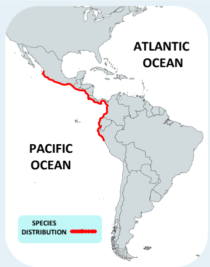

SPECIES-SPECIFIC PRIMERS					
NAME	SEQUENCE 5'-3'	SENSE	[FINAL] µM	SIZE bp	GENE
ANIN246F	TAGCTCACGCCGGAGCATCTGTC	Forward	0.1	246	COI
ANIN246R	TGGTATTTAGATTTCGGTCCGTGAGA	Reverse			

STANDARD PCR MIX	
PCR H_2O	4.1 µL
Premix 2X	5 µL
AI246F (10 µM)	0.1 µL
AI246R (10 µM)	0.1 µL
$MgCl_2$ (25mM)	0.2 µL
DNA (10-20 ng/µL)	0.5 µL
TOTAL	10 µL

DUPLEX PCR MIX	
PCR H_2O	4.1 µL
Premix 2X	5 µL
AI246F (10 µM)	0.1 µL
AI246R (10 µM)	0.1 µL
MiFish-U-F (10 µM)	0.1 µL
MiFish-U-R (10 µM)	0.1 µL
DNA (10-20 ng/µL)	0.5 µL
TOTAL	10 µL

CYCLING PROTOCOL			
STEP	TEMPERATURE °C	TIME	CYCLES
Denaturation	94	5 min	1
Denaturation	95	20 s	33
Hybridization	64	20 s	
Extension	72	20 s	
Final extension	72	5 min	1
