# Supplementary figures and images for: Development and validation of versatile species-specific primer assays for eDNA monitoring and authentication of 10 commercially important Peruvian marine species
Source: PLoS One. 2025 Jul 2;20(7):e0313181. doi: 10.1371/journal.pone.0313181 (PMC12221000; doi:10.1371/journal.pone.0313181)

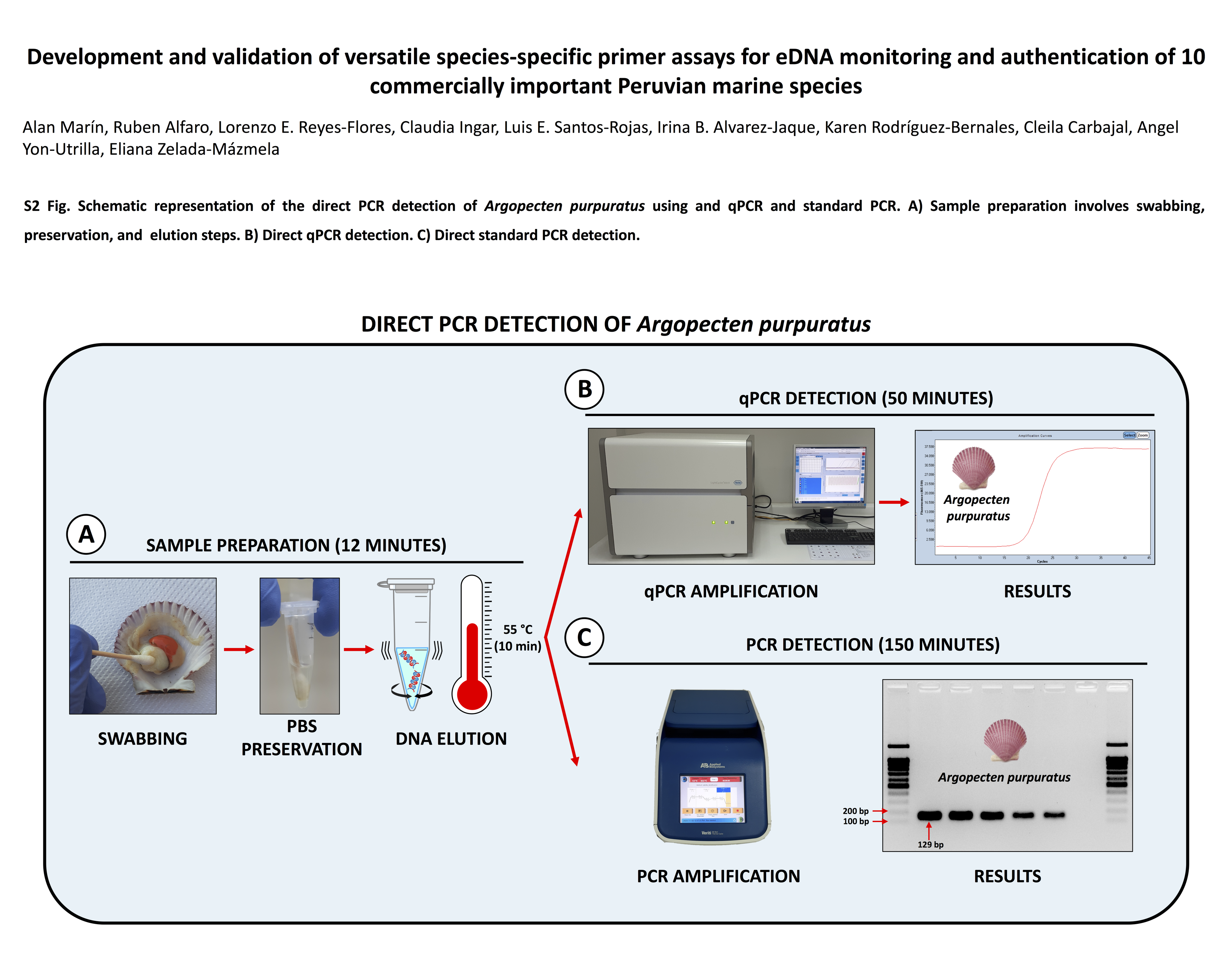

Supplement: S2 Fig — (TIF) [file pone.0313181.s005.tif]

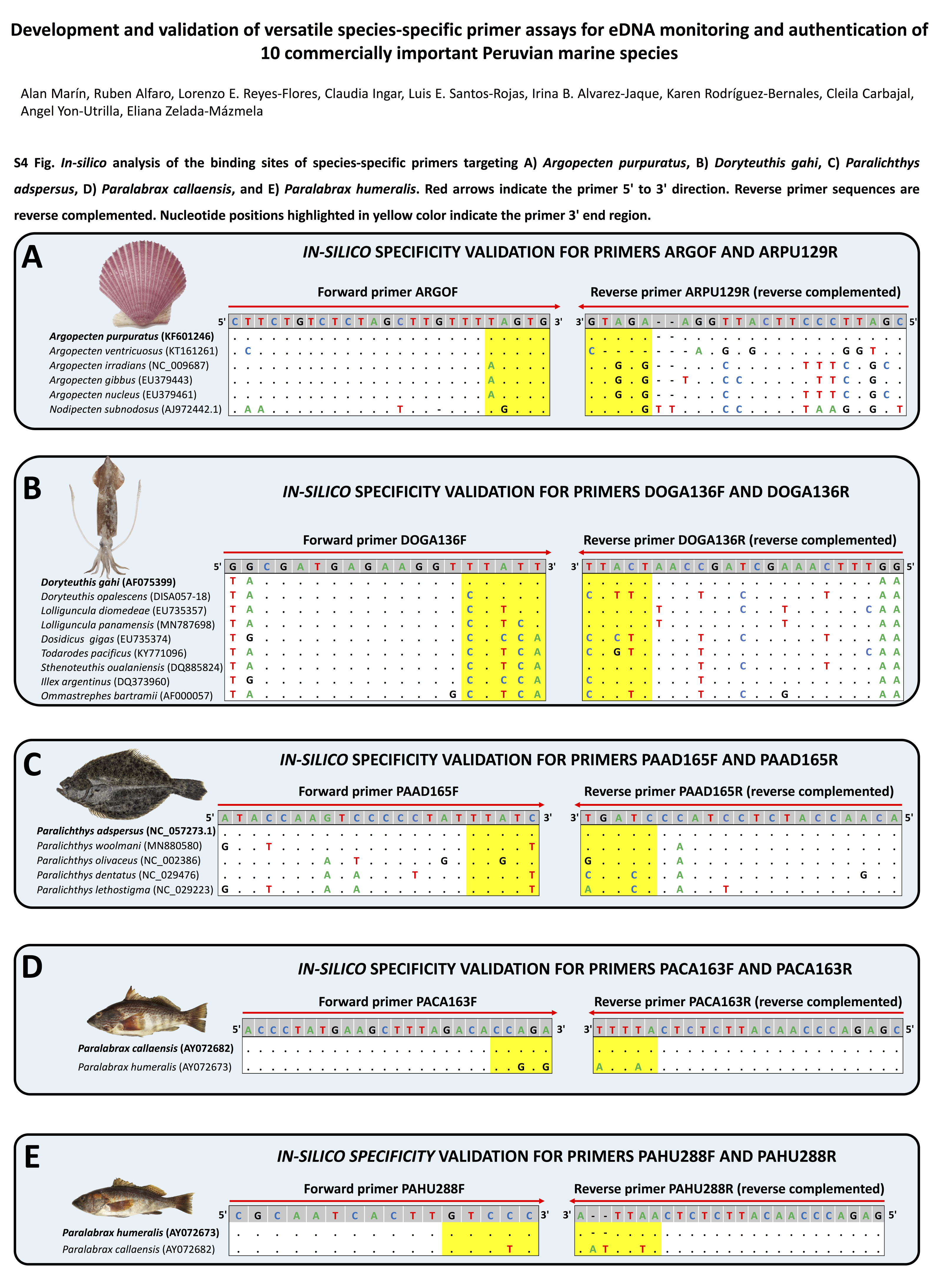

Supplement: S4 Fig — (TIF) [file pone.0313181.s007.tif]

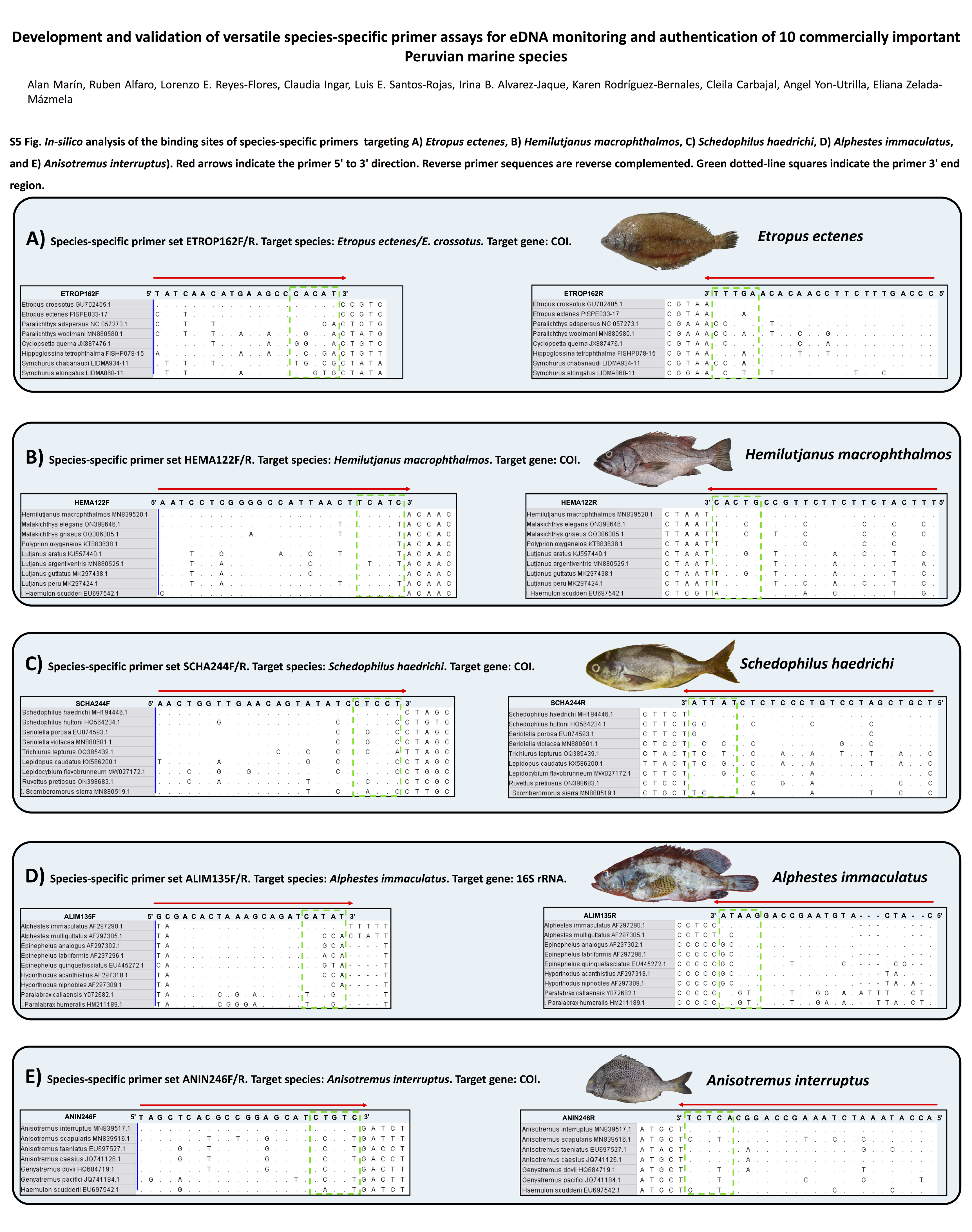

Supplement: S5 Fig — (TIF) [file pone.0313181.s008.tif]
