# Supplementary material for: Development and validation of versatile species-specific primer assays for eDNA monitoring and authentication of 10 commercially important Peruvian marine species
Source: PLoS One. 2025 Jul 2;20(7):e0313181. doi: 10.1371/journal.pone.0313181 (PMC12221000; doi:10.1371/journal.pone.0313181)

Development and validation of versatile species-specific primer assays for eDNA monitoring and authentication of 10 commercially important Peruvian marine species

Alan Marín, Ruben Alfaro, Lorenzo E. Reyes-Flores, Claudia Ingar, Luis E. Santos-Rojas, Irina B. Alvarez-Jaque, Karen Rodríguez-Bernales, Cleila Carbajal, Angel Yon-Utrilla, Eliana Zelada-Mázmela

S3 Fig. I*n-silico* analysis of the binding sites of forward primer ARGOF in 68 *A. purpuratus* sequences.

After sequence alignment, a common *Argopecten* forward primer "ARGOF" was designed based on a conserved region, whereas the specific *A. purpuratus* reverse primer namely ARPU129R was developed based on the mutations observed in a highly variable region (see panel A in S4 Fig). The in-silico specificity validation included all available GenBank sequences from the mitochondrial 16S rRNA gene from *A. purpuratus* (n=34) and sequences determined herein (n=34). The forward primer ARGOF matches perfectly to 67 sequences, while only one sequence was found to contain a single mismatch at the twelfth nucleotide from the 5' end of the primer. The reverse SSP ARPU129R had complete sequence homology with 45 *A. purpuratus* sequences (67%, haplotype "C"), exhibiting only a single mismatch at the fourth nucleotide from the 3' end in 21 sequences (30%, haplotype "D"), an additional mismatch (to those found in haplotype "D") was detected at the fifteenth nucleotide from the 3' end in two sequences (3%, haplotype "E"). Individuals containing haplotypes "D" (n=3, Genbank accessions PP087160, PP087169, PP087180) and "E" (n=1, GenBank accession PP087176) were efficiently amplified in further *in-vitro* analysis using endpoint PCR and qPCR.

A) Red arrow indicates the 5' to 3' direction. The complete matrix with all 68 DNA sequences can be found in S1 File.

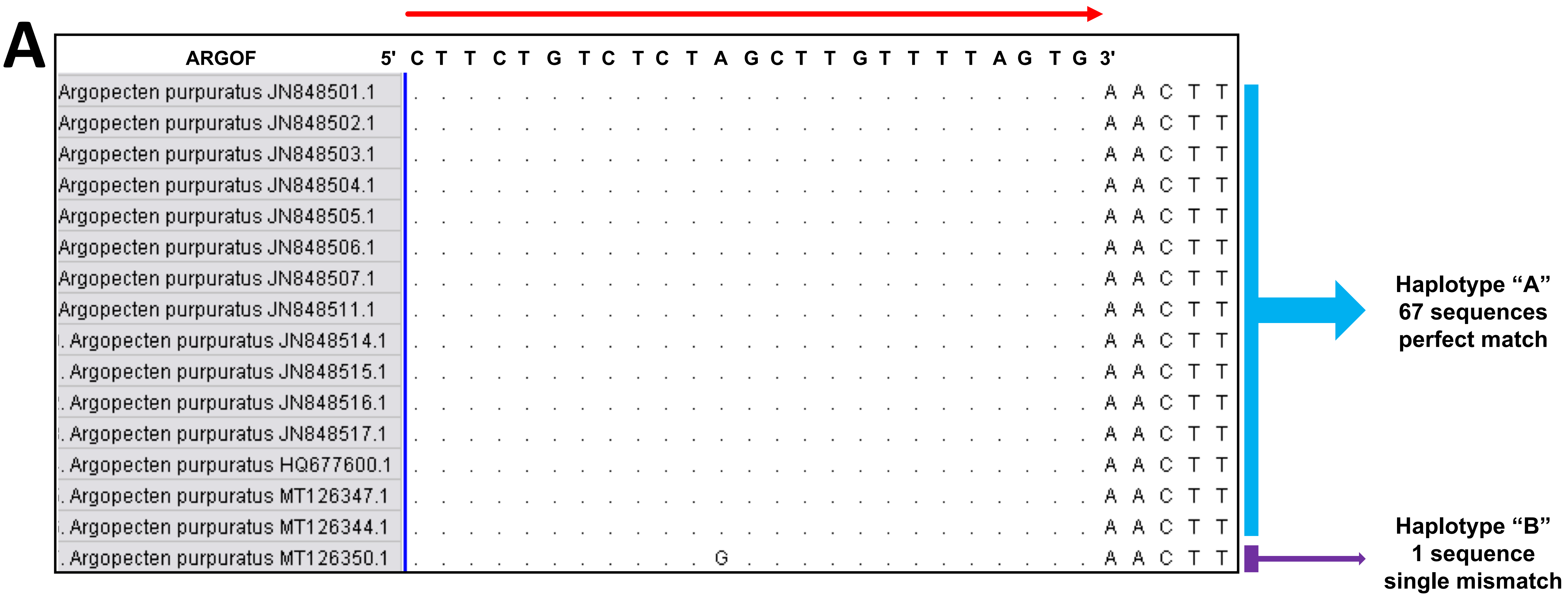

B) The primer sequence is reverse complemented and the red arrow indicates the 5' to 3' direction. The complete matrix with all 68 DNA sequences can be found in S1 File

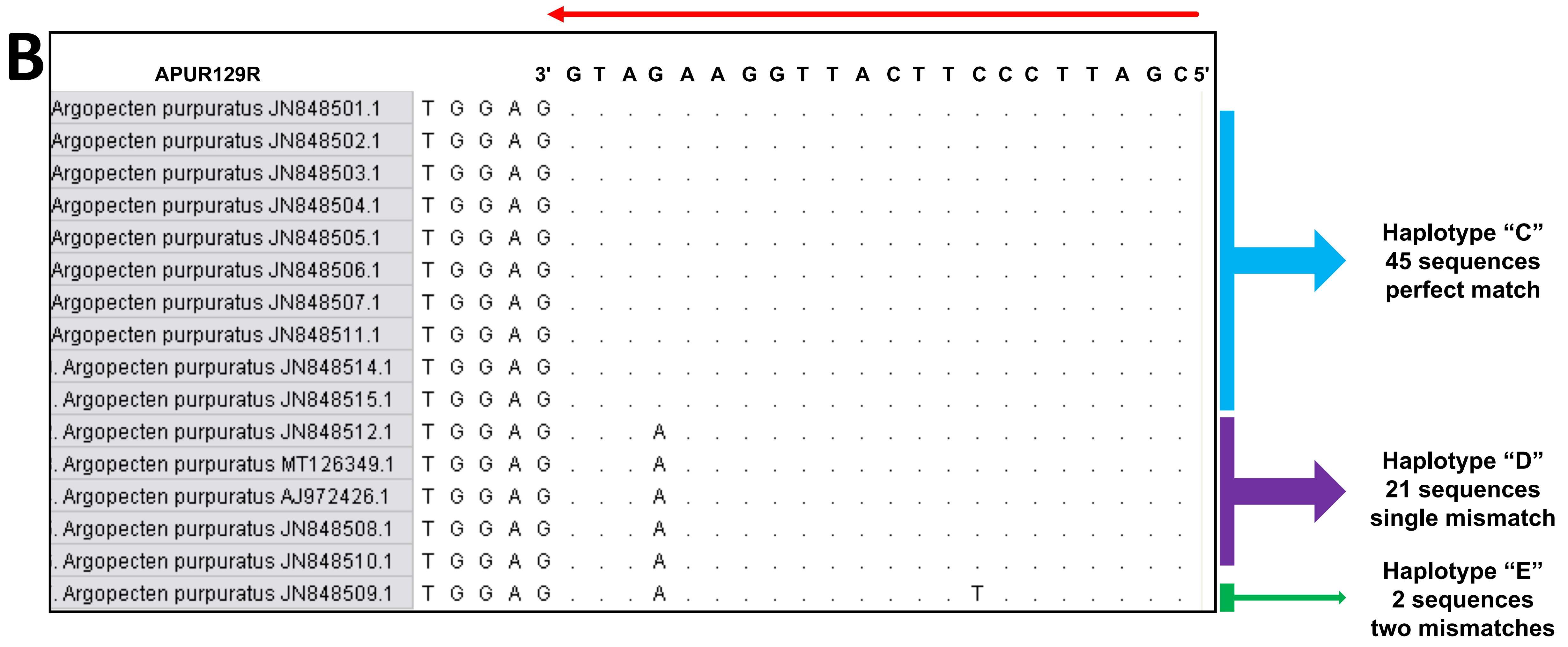

Supplement: S3 Fig — (PDF) [file pone.0313181.s006.pdf]
